# Placental Extracellular Vesicles Exhibit Reduced Neurogenic Potential Linked to Changes in Their miRNA Landscape Upon HCMV Infection

**DOI:** 10.1002/jex2.70108

**Published:** 2026-01-25

**Authors:** Charlène Martin, Hélène Martin, Mathilde Bergamelli, Lhorane Lobjois, Lucie Franco, Emma Bordes, Alexandra Benchoua, Stéphanie Balor, Diala Kantar, Etienne Coyaud, Frédéric Martins, Alexandre Favereaux, Cécile E. Malnou

**Affiliations:** ^1^ Infinity – Toulouse Institute for Infectious and Inflammatory Diseases Université de Toulouse, INSERM, CNRS Toulouse France; ^2^ CNRS, Interdisciplinary Institute for Neuroscience Université de Bordeaux, IINS, UMR Bordeaux France; ^3^ CECS, I‐STEM, AFM Corbeil Essonnes France; ^4^ METi, Centre de Biologie Intégrative Université de Toulouse, CNRS, UPS Toulouse France; ^5^ Protéomique Réponse Inflammatoire Spectrométrie de Masse (PRISM) Université de Lille, CHU Lille, Inserm U1192 Lille France; ^6^ INSERM, Neurocentre Magendie Université de Bordeaux, U1215 Bordeaux France

**Keywords:** human cytomegalovirus, neural stem cells, neurogenesis, placental extracellular vesicles, viral congenital infection

## Abstract

Extracellular vesicles (EVs) are key mediators of maternal–foetal communication, regulating placental function and foetal development through the transfer of bioactive molecules. Although placental EVs play a crucial role in placental function during pregnancy, their contribution to foetal development, notably foetal brain, remains poorly understood. Human cytomegalovirus (HCMV) is the most common virus transmitted in utero and a leading cause of infectious brain malformations. Although certain central nervous system lesions caused by HCMV are explained, the neuropathogenesis of congenital infection remains poorly understood. In this study, we demonstrate that EVs from healthy placentas promote neurogenesis. However, EVs from HCMV‐infected placentas lose this neurogenic potential, impairing differentiation and migration of neural stem cells, perturbations that may contribute to the neurodevelopmental defects observed in congenital HCMV infections. miRNA profiling revealed profound infection‐induced changes, including the incorporation of viral miRNAs and dysregulation of host miRNAs involved in neurogenesis. These findings highlight the critical role of placental EVs in foetal brain development and their contribution to HCMV neuropathogenesis.

## 1 Introduction

Placental extracellular vesicles (EVs) are key mediators of communication between the mother and developing foetus during pregnancy (Martin et al. 2022). These small membranous vesicles, ranging from 30 to 500 nm in size depending on the EV subtype, are produced by various placental cell types, primarily syncytiotrophoblasts and cytotrophoblasts (van Niel et al. 2018; van Niel et al. 2022). Placental EVs carry diverse molecular cargos, including proteins, coding and noncoding RNAs, such as microRNAs (miRNAs), which they deliver to both maternal and foetal compartments. These vesicles constitute a significant proportion of circulating EVs in maternal and foetal blood (Sarker et al. 2014; Sadovsky et al. 2020; Czernek and Duchler 2020). Recent studies have highlighted their roles in placental establishment, maternal immune tolerance toward the foetus and placental defence against viral infections (Martin et al. 2022). The composition of placental EVs, including their protein and miRNA content, is tightly regulated during pregnancy and altered under pathological conditions such as diabetes mellitus, pre‐eclampsia and intrauterine growth restriction. These changes make EVs promising biomarkers for pregnancy‐associated pathologies (Salomon and Rice 2017). Despite increasing evidence for the role of placental EVs in maternal–foetal communication, studies investigating their function in foetal development, particularly in pathological conditions such as viral infections, remain limited. This gap is notable given that placental EVs account for up to 45% of EVs in foetal blood (Miranda et al. 2018).

Human cytomegalovirus (HCMV), an enveloped DNA virus of the *Orthoherpesviridae* family, is the most common virus transmitted in utero, affecting approximately 1% of live births. It is the leading cause of infectious brain malformations and congenital deafness (Leruez‐Ville et al. 2020). The consequences of HCMV infection for newborns, particularly for subtle neurological deficits undetectable by ultrasound, are difficult to predict, and there are currently no reliable noninvasive prognostic methods (Lawrence et al. 2024; Leruez‐Ville et al. 2024). HCMV infection of the placenta facilitates viral replication and access to the foetus, including the brain (Fisher et al. 2000). Early central nervous system (CNS) lesions in the embryo or foetus can be partly attributed to HCMV infection of neural stem cells (NSCs) and/or neural precursors (Rolland et al. 2016). The direct infection of NSCs by HCMV has been shown to impact their differentiation and migration (Rolland et al. 2016; Rolland et al. 2021). However, the low proportion of infected cells in the foetal brain and the diversity of neurological sequelae caused by the infection cannot be fully explained with current knowledge. As a result, the neuropathogenesis of congenital HCMV infection remains poorly understood.

Brain development is a dynamic and finely regulated process involving cell proliferation, migration and differentiation into neurons or glial cells, underpinned by complex molecular events that require precise spatial and temporal gene regulation at both transcriptional and post‐transcriptional levels (Belmonte‐Mateos and Pujades 2021; Ossola and Kalebic 2021). Increasing evidence supports the critical role of miRNAs in neuronal development, from early neurogenesis to synaptogenesis, and in maintaining neuronal function (Cho et al. 2019; Arzua et al. 2021). Furthermore, EVs, known to carry miRNAs, can cross the blood–brain barrier, exerting significant biological effects within the CNS (Counil et al. 2025). In the foetus, where this barrier is immature, EVs are likely to cross more readily, potentially amplifying their biological impact compared to the mature adult barrier (Gamage and Fraser 2021).

In previous work, we demonstrated that EVs secreted by HCMV‐infected placental cells promote viral infection of NSCs by increasing their permissiveness to infection (Bergamelli et al. 2022). These findings aligned with observed alterations in the protein composition of placental EVs upon infection, with a profile consistent with a proviral role (Bergamelli et al. 2022; Bergamelli et al. 2021). Beyond this effect of placental EVs in facilitating HCMV infection of NSCs, and given their abundance in foetal blood, our hypothesis is that the placental EVs secreted in response to HCMV infection, through their dysregulated composition, may also initiate or contribute by themselves to the disruption of foetal brain development.

In this study, we first investigated the effects of placental small EVs (sEVs) on NSCs phenotype and identified their involvement in key aspects of neurogenesis, particularly in NSC differentiation and migration. These effects were abolished when NSCs were incubated with sEVs secreted by HCMV‐infected placental cells. Next, we analysed the miRNA content of placental sEVs and found that HCMV infection altered their miRNA profiles. Notably, we detected viral miRNAs in infected sEVs, along with host miRNA modifications, with target predictions suggesting potential interference in brain developmental pathways. These findings strongly suggest that placental EVs contribute to foetal brain development via their miRNA cargo, and that HCMV infection disrupts this function. Collectively, our study provides new insights into the neuropathogenesis of HCMV infection and highlights the critical role of placental EVs in brain development, in addition to their roles in promoting the dissemination of the virus into the foetal brain (Bergamelli et al. 2022).

## 2 Material and Methods

### 2.1 Ethics

The use of NSCs derived from human embryonic stem cells received approval from French authorities (Agence de la Biomédecine, authorisation number SASB0920178S).

### 2.2 Cell Lines

MRC5 cells (RD‐Biotech, Besançon, France) were cultured in Dulbecco's Modified Eagle Medium (DMEM with Glutamax, Gibco), supplemented with 10% foetal bovine serum (FBS, Sigma–Aldrich, Saint‐Louis, MO, USA), 100 U/mL penicillin–100 µg/mL streptomycin (Gibco) and 100 µg/mL normocin (Invivogen, Carlsbad, CA, USA).

Human invasive proliferative extravillous cytotrophoblasts (HIPEC) were obtained from Dr. T. Fournier (Inserm, Paris; Transfer agreement n°170,448) (Pavan et al. 2003). Cells were cultured with a 50/50 ratio (v/v) DMEM/F12 (Gibco, Waltham, MA, USA) medium, with the same supplementation as the MRC5. HIPECs expressed cytokeratin 7, CD9 and vimentin in accordance with the study establishing this cell line (Pavan et al. 2003), whatever the HCMV infection status (Figure S1A).

NSCs deriving from human embryonic stem cells (SA001) were cultured in N2B27 medium supplemented with brain‐derived growth factor (BDNF) (20 ng/mL), epidermal growth factor (EGF) (10 ng/mL) and fibroblast growth factor (FGF) (10 ng/mL) after double‐coating of the culture dishes with poly‐ornithine (16 mg/mL, Sigma) and laminin (2 µg/mL, Gibco) (Boissart et al. 2012). For differentiation into neurons (described in Boissart et al. 2013), the medium was supplemented with BDNF only, and laminin was systematically added (unless specified) at each medium renewal every 3 days. This population of self‐renewing late cortical progenitors spontaneously differentiates into mainly glutamatergic neurons (around 80%) and GABAergic cortical neurons in a smaller proportion (around 10%) (Boissart et al. 2013).

For luciferase reporter assay, HEK‐293 cells were seeded at 1 × 10^5^ cells per well in 12‐well plates and maintained in high‐glucose DMEM (Biowest) supplemented with 5% FBS (Gibco), 1% GlutaMAX (Thermo Fisher Scientific) and 1% penicillin–streptomycin (Thermo Fisher Scientific). Cells were incubated at 37°C in a humidified atmosphere containing 5% CO_2_.

### 2.3 Virus Production, Titration and Infection

The endotheliotropic VHL/E strain of HCMV was used in this study, kindly gifted by Dr. C. Sinzger (Ulm University, Germany). Fresh MRC5 cells were used to prepare viral stocks obtained after ultracentrifugation of cell supernatant as described elsewhere (Rolland et al. 2016). Titration of the viral stock was performed by qPCR (Mengelle et al. 2016).

### 2.4 sEV Preparation

EVs were prepared according to the MISEV guidelines (Welsh et al. 2024). All details of the preparation can be found in (Bergamelli et al. 2022). As previously described, cell culture was performed in ‘Exofree’ medium that has been depleted for EVs found in FBS. Four million HIPECs were seeded in six 150 cm^2^ flasks for each condition and infected 24 h later at a multiplicity of infection (MOI) of 10. The original medium was replaced by Exofree medium 24 h post infection (hpi). Supernatants were collected at 48 and 72 hpi and pooled for each condition (infected or not infected). Cellular debris was removed by a centrifugation at 1200 × *g* for 30 min, followed by the elimination of large EVs and viral particles at 12,000 × *g* for 30 min. sEVs were then pelleted at 100,000 × *g* for 60 min, resuspended in PBS and separated from the remaining viral particles in a discontinuous iodixanol/sucrose gradient at 100,000 × *g* during 18 h. The fractions of interest were harvested, pooled and washed in PBS before a last ultracentrifugation at 100,000 × *g* for 60 min. The pellet was resuspended in PBS, aliquoted and stored at −80°C. For uptake experiments, EVs were labelled with PKH67 (Sigma) at a 1:1000 dilution for 5 min at room temperature before performing the separation gradient. An EV‐metric analysis was realised on EV‐TRACK for the purpose of this study (Consortium et al. 2017) and indicated an EV‐metric score of 86.55%. For each sEV preparation, the absence of contaminant infectious viral particles was checked by performing an immunofluorescence against HCMV immediate early Proteins 1 and 2 on MRC5 cells incubated with sEVs (data not shown and Bergamelli et al. 2022).

### 2.5 Nanoparticle Tracking Analysis

Nanoparticle tracking analysis (NTA) was carried out to characterise the size and concentration of sEV preparations, using a NanoSight NS300 (Malvern Panalytical) equipped with a laser at 488 nm. For each preparation, three videos of 60 s each were recorded at constant temperature (20°C) and constant flux (syringe pump). The gain was fixed, and the camera level was adapted to each preparation (between 12 and 16). Data were analysed with the Software NTA 3.4 Build 3.4.4 (Malvern Instruments Ltd.).

### 2.6 Cryoelectromicroscopy

A sample of 3 µL was deposited onto glow‐discharged lacey carbon grids and placed in the thermostatic chamber of a Leica EM‐GP automatic plunge freezer, set at 20°C and 95% humidity. Excess solution was removed by blotting with Whatman No 1 filter paper for 1.8 s, and the grids were immediately flash frozen in liquid ethane at −185°C. Images were acquired on a Talos Arctica (Thermo Fisher Scientific) operated at 200 kV in parallel beam condition with a K3 Summit direct electron detector and a BioQuantum energy filter (Gatan Inc.). Energy‐filtered (20 eV slit width) image series were acquired with Digital Micrograph software at a pixel size of 0.85 Å between −1 and −1.5 µm defocus.

### 2.7 Western Blot

Western blots were carried out as described previously (Bergamelli et al. 2021) using primary and secondary antibodies described in Table S1. Membranes were visualised using the Odyssey Infrared Imaging System (LI‐COR Biosciences, Lincoln, NE, USA).

### 2.8 EV Uptake Monitoring by Flow Cytometry

To monitor EV uptake by flow cytometry, NSCs (Passages 15–18) were seeded in a 24‐well double‐coated plate (1.5 × 10^5^ cells/well) in N2B27 medium supplemented as described above. Twenty‐four hours later, different amounts of PKH67 labelled‐EVs were added directly to the wells (200, 1000, 5000 or 10,000 EVs/cell). Following another 24 h of incubation, cells were washed with PBS, trypsinised and centrifuged at 400 × *g* for 5 min to prepare for flow cytometry analysis. PKH67‐positive cells were detected using a MACSQuant Analyser 10 flow cytometer (Miltenyi Biotec) with FCS and FITC fluorescence parameters, subtracting cell autofluorescence background. Data were analysed using FlowJo (BD) software.

### 2.9 Evaluation of Gene Expression by Quantitative Real‐Time PCR (qRT‐PCR)

For neuronal differentiation, NSCs (Passages 12–14) were seeded in 12‐well plates (3 × 10^5^ cells/well) in N2B27 medium supplemented with 20 µg/mL BDNF, with addition of 2 µg/mL of laminin (Gibco). Twenty‐four hours later, and every 3 days until 28 days of differentiation, the medium was renewed with addition of 1000 EVs/cell. NSCs from the same passages were used as a control for undifferentiated cells. RNAs were extracted with the RNeasy Mini Kit (Qiagen) and reverse transcribed with the LunaScript RT SuperMix Kit (New England Biolabs) according to the manufacturer's instructions. Quantitative PCR was performed using the LightCycler PCR QC Kit (Roche) on a LightCycler 480 II system (Roche). The ΔΔCt method was used for analysing the results of the quantitative PCR, using *gapdh* and *hprt* genes as housekeeping reference genes. The different primers are listed in Table S2.

### 2.10 Indirect Immunofluorescence

Cells on coverslips were fixed with 4% paraformaldehyde (PFA) (Electron Microscopy Sciences) at room temperature for 20 min. PBS containing 0.3% Triton‐X100 (Thermofisher Scientific) was used for 10 min to permeabilise the cells, followed by 1 h incubation in blocking buffer (PBS 5% FBS). Primary antibodies were applied on coverslips for at least 3 h at room temperature (see concentrations and references Table S1) diluted in blocking buffer, before three washes in PBS. Secondary antibodies were incubated for 1 h at room temperature in blocking buffer and washed three times. DAPI was applied for 5 min before mounting in Dako S3023 fluorescence mounting medium. Upon neuronal differentiation, specifically, a pre‐fixation step was carried out with 1% PFA at room temperature for 10 min, then cells were fixed with 2% PFA for 10 min. The following steps were performed as described above. A widefield microscope (Axio Observer Z1 Zeiss) was used for image acquisition (20X NA 0.8 objective), before processing with ImageJ software.

### 2.11 Evaluation of Neurogenesis Related‐Genes Expression by RT^2^ PCR Array Analysis

NSCs (Passages 12–14) were seeded as for the qRT‐PCR, in N2B27 medium supplemented with 20 µg/mL BDNF. EVs were added 24 h later and three times a week directly in the culture medium until 17 days of differentiation. According to the manufacturer's instructions, RNAs then cDNAs were prepared using the RNeasy Mini Kit (Qiagen) followed by the RT^2^ First Strand Kit (Qiagen), respectively. The expression of 84 neurogenesis‐related genes was then performed with the RT^2^ Profiler PCR Array Human Neurogenesis (PAHS‐404ZF‐6, Qiagen), on a LightCycler480 II instrument (Roche). Data were analysed using the 2^−ΔΔCT^ method, normalised with five housekeeping reference genes (ΔCt) and with the non‐differentiated condition as reference condition (ΔΔCt).

### 2.12 Evaluation of Neurogenesis Related‐Protein Expression by Proteomics Analysis

#### 2.12.1 Neuronal Differentiation and Preparation of Samples

For neuronal differentiation, NSCs (Passages 11–12) were seeded in 6‐well plates (6 × 10^5^ cells/well) in N2B27 medium supplemented with 20 µg/mL BDNF and 2 µg/mL laminin (Gibco). Twenty‐four hours later and three times a week until 14 days of differentiation, 1000 EVs/cell were added, and the medium was renewed once a week. NSCs from the same passages were used as a control for undifferentiated cells. Cell pellets were obtained after removing the cell medium by adding 2 mL PBS/well and scraping the cells. All wells were rinsed once more with PBS, and cell suspensions were centrifuged 3 min at 350 × *g*. After another pellet washing step, the supernatant was removed, and the pelleted cells were flash‐frozen in liquid nitrogen before being stored at −80°C.

#### 2.12.2 Proteomic Analysis

Proteins were extracted from cell pellets using RIPA lysis buffer [50 mM Tris‐HCl pH 7.5, 150 mM NaCl, 1 mM EDTA, 1 mM EGTA, 1% Triton X‐100, 0.1% SDS, 1:100 protease inhibitor cocktail (Sigma–Aldrich)], sonication and centrifugation at 16,000 × *g* for 30 min to remove cell debris. Proteins were prepared with Filter Assisted Sample Preparation (FASP) (Amicon 10kDA #UFC501024, Merck Millipore) following the protocol described in (Wiśniewski et al. 2009; Wiśniewski et al. 2011). They were then digested with 40 µg/mL of trypsin (Promega) overnight at 37°C, and resulting trypic peptides were lyophilised in a SpeedVac. Dried samples were reconstituted in 60 µL of 0.1% formic acid (FA), and 20 µL from each sample was desalted using an Evotip Pure (Evosep, Denmark) according to the manufacturer's protocol. Peptides were subsequently analysed using an Evosep One liquid chromatography (LC) system coupled to a timsTOF HT mass spectrometer (Bruker Daltonics, Germany). The Evosep One system operated under the 60 samples per day (60 SPD) method with a C18 performance column (EV1109; 8 cm × 150 µm, 1.5 µm particle size) maintained at 40°C. The analytical column was connected to a fused silica emitter (10 µm inner diameter, Bruker Daltonics) integrated into a CaptiveSpray source (Bruker). Data acquisition was performed in DIA‐PASEF mode, recording spectra within an *m*/*z* range of 100–1700 and an ion mobility range of 1/*K*0 = 1.51 V·cm^−2^ to 1/*K*0 = 0.6 V·cm^−2^. Raw data were analysed using DIA‐NN software (version 1.9.2) (Demichev et al. 2020; Demichev et al. 2022). A library‐free workflow was used to search against the UniProt reviewed *Homo sapiens* database (September 2024, 20,420 entries). Search parameters allowed up to two missed cleavages for trypsin digestion, with methionine oxidation specified as a variable modification. Peptides were filtered using a length range of 7–30 amino acids, a precursor charge range of 2–4, a precursor *m*/*z* range of 300–1300 and a fragment ion *m*/*z* range of 100–1700. False discovery rates (FDRs) were set to 1% at both the protein and peptide levels. ‘Match between runs’ was enabled, and the quantification strategy was set to ‘robust LC (high accuracy)’.

Biological triplicates were performed for each condition, and relative abundances of identified proteins [label‐free quantification (LFQ)] were compared between conditions when at least two experimental LFQ values were measured out of three biological replicates for a given ID and condition. To allow for statistical comparison, missing values were treated as follows: if one value was missing, it was replaced by the mean value of Replicate 1 and Replicate 2; if a protein was detected in one or zero replicate, missing values were replaced by the minimal value found among all proteins present in the sample of interest. A *p* value was determined with a Student's *t* test by comparing LFQ values between two conditions. Differences were considered significant with a log2 FC > 1 or < −1, and a *p* value < 0.05. The mass spectrometry proteomics data have been deposited to the ProteomeXchange Consortium via the PRIDE partner repository with the dataset identifier PXD060921 (webpage: https://www.ebi.ac.uk/pride/login; username: reviewer_pxd060921@ebi.ac.uk; pw: 7jHiQV7UrU2U) (Perez‐Riverol et al. 2022). They are available in the Supporting Information Proteomic Data document.

### 2.13 Migration Assays

NSCs were seeded in 48‐well plates (3 × 10^5^ cells/well). Forty‐eight hours later, a scratch was performed with a 10 µL pipet tip on the confluent cell layer. Cells were washed twice with 150 µL of PBS before addition of fresh medium supplemented with laminin (2 µg/mL). EVs were added directly into the well when necessary. Image acquisition was performed with OPERA Phenix (PerkinElmer) with the 20X air objective. All scratch edges were acquired at different time points according to the experiment. Images were analysed on ImageJ (Suarez‐Arnedo et al. 2020). Areas of interest were cropped and smoothed, and parameters were adapted for each picture for allowing the model to fit to the edges observed, and the surface area of the scratch was measured. Results were expressed as recovery percentages, calculated by the ratio between the area observed at the final time point and the area observed at time 0.

### 2.14 Quantitative miRNA Analysis

#### 2.14.1 RNA Extraction

The Direct‐Zol RNA MiniPrep kit (R2060, Zymo) was used to extract RNAs according to the manufacturer's instructions, using whole EV preparations. Total RNAs were eluted in 12 µL ultra‐pure water. The concentration of RNA was measured by spectrophotometry (NanoDrop 2000, ThermoFisher), and RNA quality was checked using Lab Chip GX touch HT (PerKin Elmer) and RNA chip (Xmark – High sensitivity).

#### 2.14.2 Small RNA Libraries Preparation and Sequencing

The libraries were synthesised from 100 ng RNA for each sample, following the manufacturer's instructions (NEXTFLEX Small RNA Seq v3, PerkinElmer). Briefly, 3′ adenylated adaptors, followed by 5′ adaptors, were sequentially added to RNA strands. RNA samples were then reverse transcribed using the 3′ adaptors as the template for the RT primer. Finally, cDNAs were amplified by PCR. The quality of sequencing libraries was analysed by capillary gel electrophoresis (Lab Chip GX touch HT, PerkinElmer). After quantification of individual libraries by qPCR using the NEB Next library quant kit (containing primers which target the P5 and P7 Illumina adaptor sequences, New England BioLabs), all the samples were pooled in an equimolar manner before sequencing at a concentration of 750 pM. miRNA sequencing was performed using Nextseq 2000 Illumina (single‐end 1 × 50 bp) at the PGTB facility (INRAE – Pierroton), with an average of 13 million reads per sample. The sequences are available on the SRA database (NCBI) (reference PRJNA1129057).

#### 2.14.3 Identification of Human and HCMV miRNAs by Bioinformatic Analysis

To obtain clean reads, the adaptor sequences from the library preparation and the 4 random nucleotides inserted between the adaptor sequence and the sequence of the miRNAs were removed, using the Cutadapt tool. The *H. sapiens* genome reference was downloaded from UCSC Genome Browser assembly ID:hg38. All bioinformatic analyses were performed using the miRPro program (Shi et al. 2015), which uses the main algorithm of the miRDeep2 software (Friedländer et al. 2012). The main advantage of miRPro compared to miRDeep2 is that it proposes consistent and unified names for novel precursors and their mature miRNAs in all libraries of the samples. The reads issued from the sequencing were aligned against the genome of reference by using Novoalign (Li and Homer 2010). Sequences matching known human small RNAs such as rRNA, scRNA, snoRNA, snRNA and tRNA or degradation fragments of mRNAs were excluded in further analyses, and sequences that perfectly matched the human genome along their entire length and recognised as miRNAs were subjected to subsequent analyses. To detect HCMV miRNAs, we downloaded all HCMV miRNA precursors, pre‐mature and mature sequences from miRbase (Kozomara et al. 2019) and we used miRPro as previously described.

#### 2.14.4 Differential Expression of miRNA

A differential expression analysis was performed on R to detect miRNA expression changes between the two conditions, by using the DESeq2 Bioconductor's package 1.40.2 (Love et al. 2014). All duplicates were removed (plyr package), and miRNAs from infected or uninfected HIPECs were paired when coming from the same preparation. Results and normalised counts were then extracted. miRNAs were considered differentially expressed when the *p* value was <0.05 and the log2 fold change (log2 FC) was >2 or <−2. hsa‐miRNA 3690 was manually removed from the analysis since it was identified only in one replicate. All scripts and files used are available on GitHub (https://github.com/CMart217/R‐script—differential‐expression‐of‐miRNA).

#### 2.14.5 miRNA Target Prediction

Targets of human miRNAs of interest (i.e., miRNAs differentially expressed in NI vs. HCMV condition with a *p* value <0.05 and a log2 FC >2 or <−) were analysed using the Qiagen Ingenuity Pathway Analysis software (IPA, Qiagen), by running the miRNA target filter tool. Only the ‘experimentally proved’ target mRNAs found in human databases were selected for further analyses. The entire list of mRNAs was then used to investigate the ‘Disease and function’, ‘Canonical pathways’ and ‘Upstream regulators’ via core expression analysis on IPA. For viral miRNAs, the miRDB database was used to predict potential human mRNA targets, since HCMV miRNAs are not implemented in the IPA knowledge database (Chen and Wang 2020). All targets with a predicted score higher than 50 were collected and implemented on IPA to perform a core analysis.

#### 2.14.6 Integrated Analysis of miRNA, Transcriptomic and Proteomic Dataset

miRNA sequencing, RT^2^ array and proteomic results were combined in an integrated analysis. Differentially expressed miRNAs (i.e., *p* value <0.05, log2 FC >2 or <−2) were selected, and their experimentally validated targets were retrieved from miRTarBase (v9.0) while predicted targets were obtained from TargetScan (v8.0). These target lists were cross‐referenced with the sets of dysregulated mRNAs resulting for the RT^2^ and proteomic data. For each interaction, the direction of regulation was examined (miRNA upregulated and target downregulated, or conversely) to identify inverse expression patterns consistent with miRNA‐mediated repression. All analyses were performed in R (v4.5.0) using the packages dplyr, readr and biomaRt. The script and files are available on GitHub (https://github.com/CMart217/Scripts_EV_pla_differential_expression_of_miRNA.git).

### 2.15 Luciferase Reporter Assays

The pmirGLO Dual‐Luciferase miRNA Target Expression Vector (Promega) was used to quantitatively assess miRNA‐mediated translational repression of predicted target genes. Briefly, the 3′UTR region of the glucose‐6‐phosphate isomerase (GPI) target gene containing the putative hsa‐miR‐486‐5p binding site (Table S3) was cloned downstream of the *Firefly* luciferase reporter gene (luc2). For normalisation, the pmirGLO vector includes a constitutively expressed *Renilla* luciferase cassette (hRluc‐neo), which is not affected by miRNA targeting.

To generate luciferase reporter constructs, two complementary oligonucleotides (∼30 bp) encompassing the miRNA seed‐binding sequence were annealed and inserted into the pmirGLO vector. To verify miRNA‐specific hybridisation and silencing, mutated reporter constructs were generated by replacing the seed‐matching region with its complementary sequence, thereby preventing miRNA binding. For miRNA expression, genomic regions of approximately 350 bp spanning each miRNA locus were amplified and cloned into pcDNA3.1(+). All plasmids used in this study are listed in Table S4.

For the luciferase assay, HEK‐293 cells were cotransfected with the luciferase reporter plasmids and the corresponding miRNA expression vector using X‐tremeGENE HP DNA Transfection Reagent (Sigma–Aldrich). After 24 h, luciferase activities were quantified using the Dual‐Luciferase Reporter Assay System (Promega, #E1910) on a VICTOR Nivo microplate reader (Revvity). Luciferase data were analysed by calculating the ratio of *Firefly* to *Renilla* luciferase activity for each sample. Outlier detection was performed using *z* scores, computed as *z* = (value − mean)/SD, where the mean and standard deviation (SD) were calculated separately for each experimental condition. Samples with a *z* score greater than 1.5 were excluded from subsequent analyses. Luciferase activity was then quantified as a function of the proportion of mutated reporter construct, and the resulting values were normalised to the difference in signal between samples transfected with or without hsa‐miR‐486‐5p.

### 2.16 Statistics

Statistical analyses were performed using GraphPad Prism (v10) software. For each data set, a normality test was performed, and the appropriate statistical test was run consequently to compare data as indicated in the figure legends.

## 3 Results

### 3.1 Preparation and Characterisation of Placental Small EVs

**FIGURE 1:**
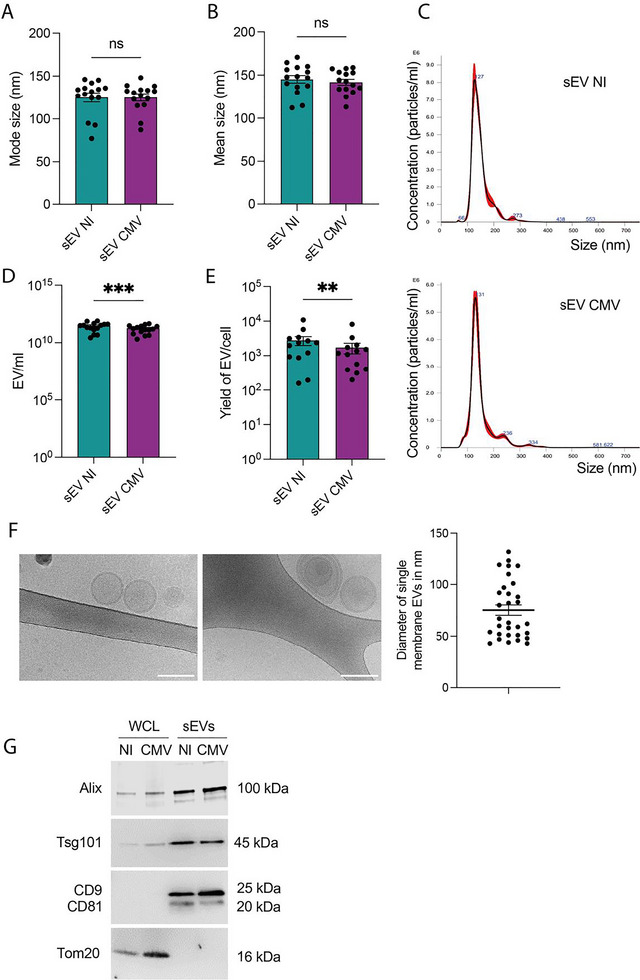
Characterisation of the sEV preparations. (A, B) Comparison of mode (A) and mean (B) size of EVs prepared from uninfected (sEV NI) or infected (sEV CMV) HIPECs. Histograms represent the mean ± SEM of 15 independent experiments. EV preparations (NI vs. CMV) were performed using the same batch of HIPECs, which were infected or not, for each independent experiment. ns: non‐significant by paired *t* test. (C) Graphs representing the typical distribution of EV size measured by nanoparticle tracking analysis performed on sEV NI (left panel) or sEV CMV (right panel). (D, E) Yield of sEVs recovered from uninfected (sEV NI) or infected (sEV CMV) HIPECs for total preparation (D) or per cell (E). Histograms represent the mean ± SEM of 15 independent experiments. ***p < 0.001 by paired *t* test. (F) Cryoelectromicroscopy images of sEVs prepared from uninfected HIPECs. Scale bar = 100 nm. On the right is indicated the diameter (nm) measured on single membrane vesicles (mean ± SEM, representing about 70% of the EVs in the preparation). (G) Western blot showing the enrichment of several proteins in sEV preparation compared to whole cell lysates (WCLs). Tom20 is a non‐EV control (mitochondrial protein). EV, extracellular vesicle; HIPEC, human invasive proliferative extravillous cytotrophoblast; sEV, small EV.

To investigate the impact of placental EVs on NSC phenotype, sEVs were prepared from the cytotrophoblastic HIPEC cell line, either uninfected or infected with HCMV. Before proceeding to EV preparations, we verified that the cells were permissive for HCMV (Figure S1B) and remained viable for the duration of the experiment (Figure S1C, D), in agreement with our previous findings (Bergamelli et al. 2022). NTA analyses revealed that all sEV preparations displayed size distributions consistent with expected values for sEVs, with a mode size of 125 nm for sEVs derived from both uninfected and infected HIPECs. The mean sizes were 145 and 142 nm, respectively, with no significant differences due to the infection status of the cells (Figure 1A–C). The yield of sEVs recovered from uninfected HIPECs was slightly higher than that from infected cells, both in total preparation (3.1 × 10^11^ vs. 1.8 × 10^11^ sEVs/mL, respectively; Figure 1D) and when normalised per cell (2.8 × 10^3^ vs. 1.7 × 10^3^ sEVs/cell, respectively; Figure 1E). Cryoelectron microscopy confirmed the expected ultrastructure of the vesicles, showing predominantly single‐membrane vesicles in our preparations (Figure 1F). Finally, western‐blot analyses confirmed the expected enrichment of the CD9 and CD81 tetraspanins, as well as of the ESCRT protein Tsg101 and ESCRT‐associated protein Alix. They also confirmed the absence of contaminant mitochondrial protein Tom20 in sEV preparations when compared to whole cell lysates (Figure 1G). Taken together, these elements indicate that our preparations present the typical features of sEVs with a high purity, using a robust experimental procedure (EV‐metric of 86.55%).

### 3.2 Placental sEVs Are Captured by NSCs and Do Not Impact Their Growth and Proliferation

Since placental EVs account for up to 45% of foetal blood EVs, we examined their potential impact on NSC phenotype. First, we investigated whether NSCs could capture fluorescently labelled placental EVs, prepared from uninfected or HCMV‐infected HIPECs. The proportion of fluorescent NSCs increased proportionally with the dose of EVs applied, with no significant difference observed between sEVs originating from uninfected or HCMV‐infected HIPECs (Figure S2). This indicates that any potential subsequent phenotypic effects of EVs on NSCs will likely not result from differences in their interaction with NSCs depending on their HCMV status (i.e., surface attachment or internalisation, as this experiment cannot distinguish between these two scenarios).

**FIGURE 2:**
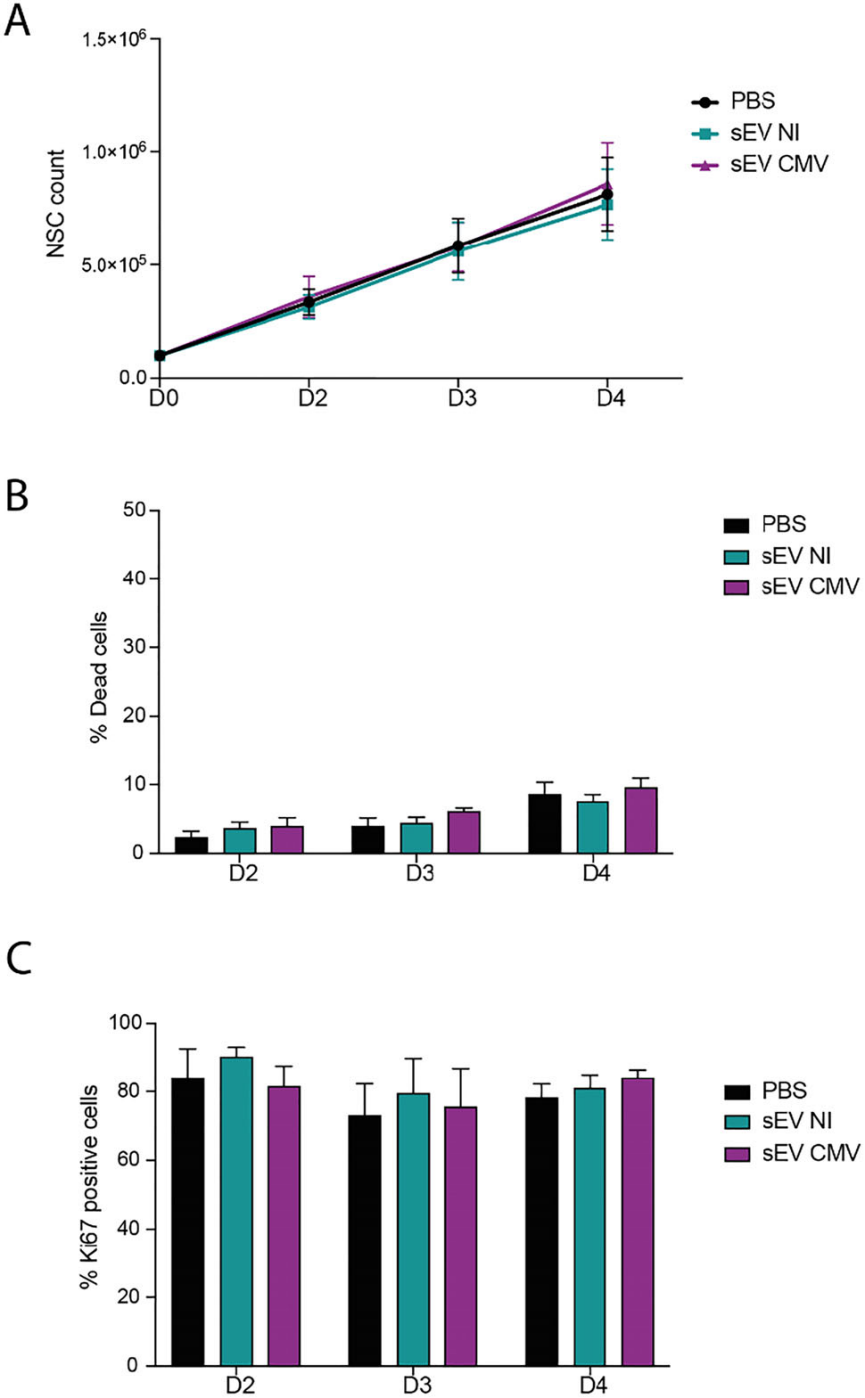
Placental sEVs do not impact growth, mortality and proliferation of NSCs. (A) Impact on growth. The *y* axis represents the NSCs count at different days (D) post‐seeding, indicated in the *x* axis. 10^5^ cells were seeded at D0 for all conditions, then the medium was renewed at D1 either with filtered PBS (black line), sEVs from uninfected HIPECs (sEV NI; green line) or sEVs from HCMV‐infected HIPECs (sEV CMV; purple line) with an amount of 1000 EVs/cell. *N* = 7. (B) Impact on mortality. The *y* axis represents the percentage of dead NSCs in the culture, evaluated by trypan blue exclusion, at different days (D) post‐seeding, indicated in the *x* axis. The experiment was performed as in (A). *N* = 7. (C) Impact on proliferation. Cells were seeded on coverslips at D0, sEVs or PBS were added at D1 as before and an anti‐Ki67 immunofluorescence was performed. The *y* axis represents the percentage of Ki67‐positive NSCs at different days (D) post‐seeding, indicated in the *x* axis. *N* = 4. All results are represented as mean ± SEM. For each experiment, a two‐way ANOVA test was performed and indicated no effect for sEV treatment. EV, extracellular vesicle; HCMV, human cytomegalovirus; HIPEC, human invasive proliferative extravillous cytotrophoblast; NSC, neural stem cell; sEV, small EV.

We next investigated whether placental sEVs could influence NSC growth and proliferation (Figure 2), by incubating NSCs with either PBS or sEVs derived from uninfected or HCMV‐infected HIPECs. We applied 1000 EVs/cell, which we consider physiologically relevant, consistent with our recovery yield of approximately 2000 EVs/cell, using the highly pure but low‐yield preparation method combining differential and density gradient ultracentrifugation. Cell growth, death and proliferation were monitored by assessing (i) total cell numbers, (ii) cell death via trypan blue exclusion and (iii) cell proliferation using anti‐Ki67 immunofluorescence. No significant differences were observed between the conditions for any of the parameters studied (Figure 2A–C), even at higher doses of EVs, up to 10,000 EVs per cell (Figure S3). Additionally, this confirmed that sEVs from either condition did not exhibit any major toxicity toward NSCs. For the following experiments, we kept the dose of 1000 EV/cell.

These results indicate that placental sEVs, whether derived from uninfected or HCMV‐infected HIPECs, are efficiently captured by NSCs and do not influence their growth, proliferation or viability under the tested conditions.

### 3.3 HCMV Infection Impairs the Ability of Placental sEVs to Promote NSC Differentiation

**FIGURE 3:**
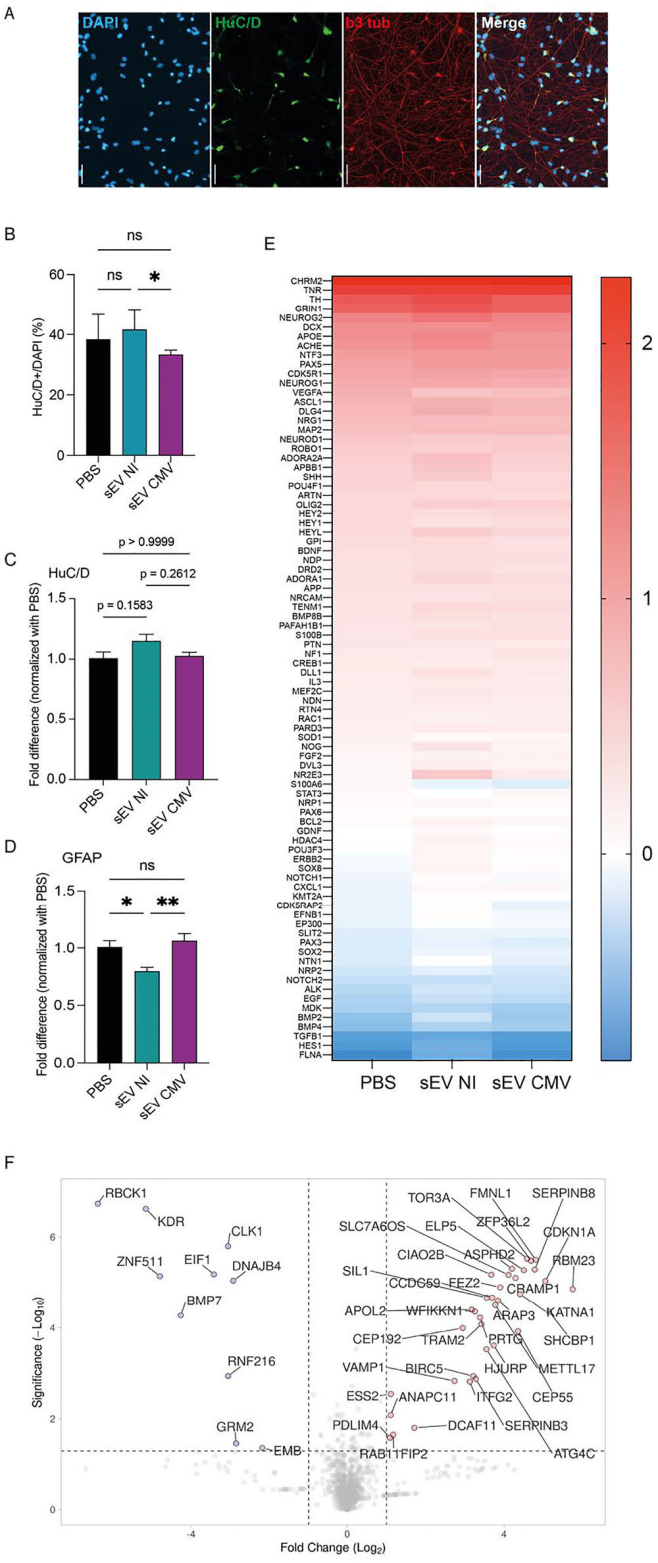
HCMV infection impairs the ability of placental sEVs to promote NSC differentiation. NSCs were seeded at Day 0 in the presence of BDNF to trigger differentiation. Three times a week, NSCs were incubated with PBS or 1000 sEVs/cell prepared from uninfected (NI) or HCMV‐infected (CMV) HIPECs. (A) Indirect immunofluorescence realised at 10 days post‐differentiation on PBS‐treated NSCs (DAPI: blue; HuC/D: green; β3 tubulin: red). Images are representative of three independent experiments. Scale bar = 50 µm. (B) Quantification by indirect immunofluorescence of the percentage HuC/D positive cells among total NSCs upon 10 days of differentiation. Histogram representing the mean ± SEM of three independent experiments. For each experiment, three independent fields were counted for HuC/D and DAPI positive cells, representing around 1000 cells per condition and per experiment. A one‐way ANOVA test indicates a *p* value of 0.0227 between the different conditions. A Bonferroni post‐hoc test was realised, which results are indicated above the histogram (*p value < 0.05). (C, D) Quantification by qRT‐PCR of HuC/D (C) or GFAP (D) expression upon respectively 7 and 14 days of differentiation, normalised by the non‐treated (PBS) condition. Histograms representing the mean ± SEM of six independent experiments. A one‐way ANOVA followed by a Bonferroni post‐hoc test was realised, which results are indicated above the histograms (*p value < 0.05; **p value < 0.01). (E) Heat‐map representing the result of an RT2‐profiler PCR array ‘Neurogenesis’ analysis realised at 17 days of differentiation. The red/blue colour gradient indicates the log fold induction of gene expression (genes listed at the left of the heat‐map) compared to undifferentiated cells. *N* = 4. A two‐way ANOVA was realised and indicates a *p* value < 0.0001 between the different treatments. (F) Volcano‐plot of mass spectrometry analyses representing differences in mean protein abundances in NSCs after 14 days of differentiation in the presence of sEVs from HCMV‐infected HIPECs versus uninfected HIPECs. Proteins exhibiting significant differences between the two conditions are represented by circles. Red: over‐represented proteins; Blue: under‐represented proteins (*p* value ≤ 0.05 and log2 fold change ≥ 1 or ≤ −1; *N* = 3). EV, extracellular vesicle; HCMV, human cytomegalovirus; HIPEC, human invasive proliferative extravillous cytotrophoblast; NSC, neural stem cell; sEV, small EV.

To evaluate whether placental EVs could contribute by themselves to the disruption of foetal brain development during HCMV congenital infection, we next investigated their ability to influence NSC differentiation into neurons. NSCs were cultured in the presence of brain‐derived neurotrophic factor (BDNF), a treatment known to drive neurogenesis in vitro (Rolland et al. 2016). Differentiation into neurons was characterised by changes in the morphology and phenotype of the cultures, which displayed HuC/D positive somas and β3‐tubulin positive axonal and dendritic projections, observed as early as 10 days post‐differentiation (Figure 3A). RT‐qPCR experiments confirmed that our experimental conditions ensured the progressive loss of stemness markers such as Sox2 and nestin, which coincided with an increase in the expression of neuronal markers, including HuC/D and MAP2 (Figure S4). Although NSCs could be differentiated for up to 28 days, their commitment to the neuronal lineage was not exclusive, as some cells differentiated into glial cells expressing GFAP (Figure S4). Therefore, to avoid confounding factors linked to glial cell overgrowth, we focused on earlier time points (≤17 days) to evaluate the impact of placental sEVs on differentiation.

We then analysed the effect of placental sEVs on NSC differentiation into neurons. Although treatment with sEVs from uninfected HIPECs produced a slight enhancement in HuC/D expression compared to PBS‐treated NSCs, incubation with sEVs from HCMV‐infected HIPECs resulted in a significant reduction (approximately 20%) of the proportion of HuC/D‐positive cells observed by immunofluorescence (Figure 3B), with a similar trend when assessed by qRT‐PCR (Figure 3C). Concomitantly, sEVs from uninfected HIPECs inhibited NSC differentiation into glial cells compared to PBS‐treated controls when monitoring the GFAP expression (Figure 3D). This inhibition was abolished when NSCs were treated with sEVs from HCMV‐infected HIPECs.

To further explore the impact of sEVs on NSC differentiation, we performed a simultaneous quantification of 84 neurogenesis‐related genes in NSCs undergoing differentiation for 17 days, using an RT^2^ Profiler assay. Differentiation of PBS‐treated NSCs induced a specific pattern of gene expression upon time (Figure 3E). Remarkably, consistent with our morphological and RT‐qPCR analyses, treatment with sEVs from uninfected HIPECs significantly modified this pattern, whereas sEVs from HCMV‐infected HIPECs restored a gene expression profile similar to PBS‐treated NSCs. Specifically, 14 genes were upregulated and 3 downregulated by sEVs from uninfected HIPECs compared to either PBS‐treated NSCs or those treated with sEVs from HCMV‐infected HIPECs (Figure S5A). Ingenuity pathways analysis (IPA, Qiagen) of these genes highlighted their involvement in the NOTCH signalling pathway, which plays a critical role in embryogenesis, brain development and neural cell fate determination (Lasky and Wu 2005; Zhou et al. 2010) (Figure S5B). Collectively, these results suggest that placental sEVs promote NSC differentiation into neurons under physiological conditions, a process disrupted when sEVs are produced by HCMV‐infected placenta.

To gain a comprehensive and unbiased assessment of the impact of sEVs on NSC differentiation, we performed a proteomic analysis of NSCs upon differentiation, following incubation with sEVs from either uninfected or HCMV‐infected HIPECs. Differentiation induced significant proteomic changes compared to undifferentiated NSCs (PBS‐treated NSCs), with the abundance of 1584 proteins significantly varying (Figure S6A). As expected, IPA analysis identified neuronogenesis‐related terms such as ‘Synaptogenesis’, ‘GABA receptor’, and ‘Axonal guidance’ (Figure S6B). When differentiation was carried out in the presence of placental sEVs, the proteome changes globally mirrored those seen in PBS‐treated NSCs upon differentiation, although some proteins were differentially expressed depending on whether sEVs were derived from uninfected or HCMV‐infected HIPECs.

Specifically, 35 proteins were upregulated and 10 downregulated in NSCs treated with sEVs from HCMV‐infected cells compared to those treated with sEVs from uninfected cells (Figure 3F, Table S5). Although the relatively small number of differentially expressed proteins precluded large‐scale regulatory network analyses, individual proteins of interest were identified. For example, GRM2 (metabotropic glutamate receptor 2), a critical receptor in glutamatergic cortical neurons, was found downregulated upon treatment with sEVs from HCMV‐infected HIPECs (Brazel et al. 2005). Similarly, BMP7, known to promote neuronal differentiation (Karunungan et al. 2023; Li et al. 2024; Zhang et al. 2021; Zhang et al. 2023), was also downregulated. Conversely, some proteins were upregulated in the HCMV condition, such as CDKN1A (cyclin‐dependent kinase inhibitor 1), which regulates NSC proliferation (Marqués‐Torrejón et al. 2013; Luyckx et al. 2018; Han et al. 2017), and CEP55 (centrosomal protein of 55 kDa) implicated in neural cell division (Frosk et al. 2017; Little et al. 2021; Yanagi et al. 2019).

These results reinforce the notion that placental sEVs promote NSC differentiation into neurons, a process that is lost upon HCMV infection, which may contribute to the neuropathogenesis associated with congenital HCMV infection.

### 3.4 HCMV Infection Impairs the Ability of Placental sEVs to Control NSC Migration

During foetal brain development, migration is a crucial step, as it ensures the proper positioning of neuronal progenitors before differentiation occurs. Severe developmental abnormalities associated with congenital HCMV infection, such as lissencephaly and polymicrogyria, are considered as neural cell migration disorders (Rolland et al. 2021; Guerrini and Parrini 2010; Kriegstein and Noctor 2004). We therefore investigated whether EVs could influence the migration of undifferentiated NSCs.

**FIGURE 4:**
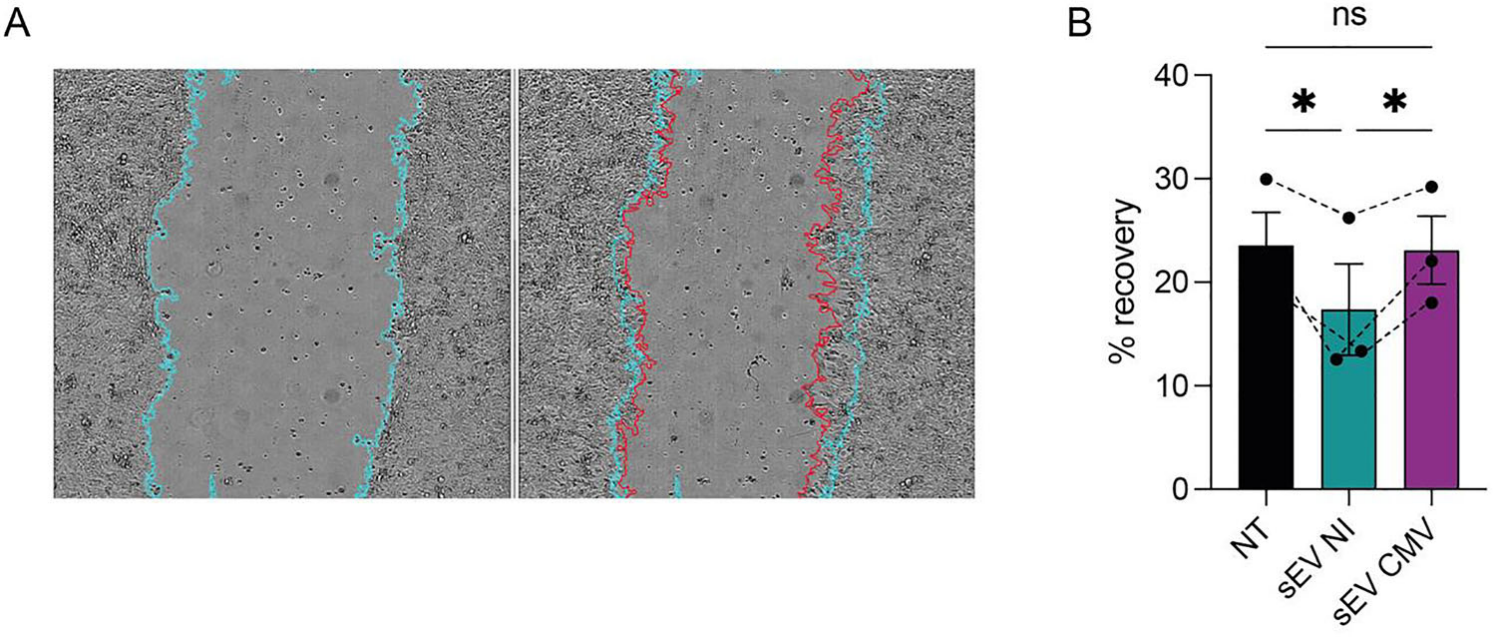
HCMV infection impairs the ability of placental sEVs to control NSC migration. (A) NSCs were seeded to reach confluency. They were incubated with PBS or placental sEVs during 2 h before performing a scratch on the cell monolayer (initial time; left picture). At the final time (6 h post‐scratch; right picture), percentages of recovery were calculated by subtracting the surface devoid of cell at the initial time (blue line) and at the final time (red line). (B) Histogram representing the mean ± SEM of three independent experiments. A one‐way ANOVA test indicates a *p* value of 0.0231 between the different conditions. A Tukey post‐hoc test was realised, which results are indicated above the histogram (*p value < 0.05). EV, extracellular vesicle; HCMV, human cytomegalovirus; NSC, neural stem cell; sEV, small EV.

NSCs were seeded to confluency, incubated with sEVs, and a controlled scratch was performed on the cell monolayer (Figure 4A). Complete recovery of the wound was observed after 24 h across all conditions (data not shown), confirming the strong migratory potential of NSCs but precluding the detection of differences induced by placental EVs at this time. Consequently, the experiment was repeated over a shorter timeframe (6 h) to better capture differences in migration while minimising the impact of cell proliferation, which was expected to be negligible based on our previous data.

Interestingly, NSCs incubated with sEVs derived from uninfected HIPECs exhibited a significant reduction in migration compared to mock‐treated NSCs (Figure 4B). In contrast, incubation with sEVs prepared from HCMV‐infected HIPECs significantly accelerated NSC migration, achieving recovery rates similar to those of PBS‐treated cells. This suggests that incubation with EVs from HCMV‐infected cells makes neural progenitors more prone to migration compared to EVs from uninfected cells, which could contribute to migration abnormalities observed in congenital HCMV infection. Additionally, this could potentially divert them from their differentiation capacity, in line with our previous findings.

### 3.5 miRNA Content of Placental Small EVs Is Altered Upon HCMV Infection

**FIGURE 5:**
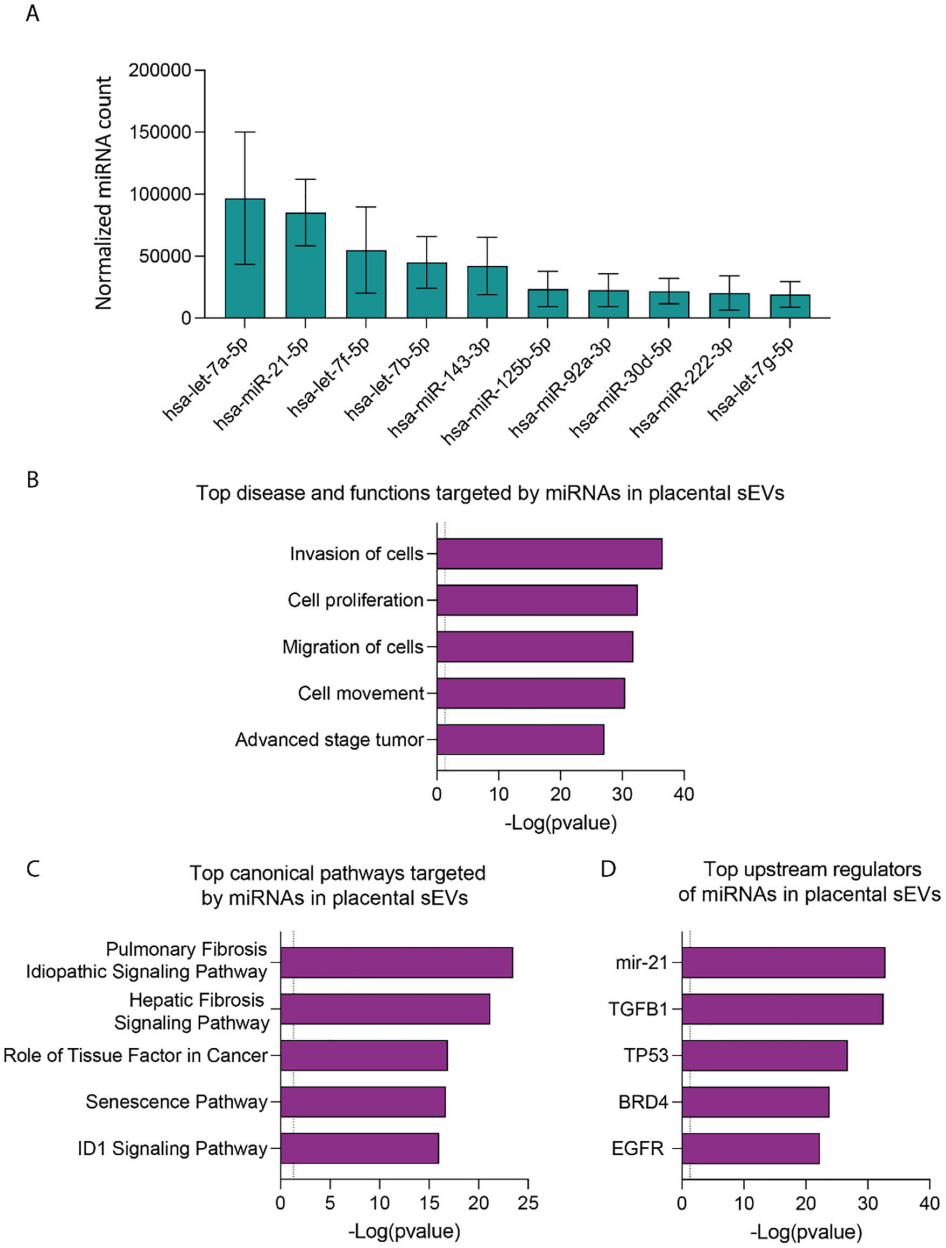
miRNA composition of placental small EVs. (A) Top 10 cellular miRNAs present in sEVs prepared from placental cells. Histogram represents the mean ± SEM of miRNA counts found in sEVs upon normalisation (*N* = 5). (B, C) QIAGEN Ingenuity Pathway Analysis realised on the cellular targets (‘experimentally proved’ in mRNA target filter) of the 10 highest expressed miRNAs found in placental sEVs. Histograms represent the Top 5 ‘Disease and functions’ categories (B), Top 5 ‘Canonical pathways’ (C) and Top 5 ‘Upstream regulators’ that may be impacted by the mRNA targets, ranged according to their *p* value (redundant terms were manually removed from the initial list). The dotted line represents the 0.05 *p* value threshold. EV, extracellular vesicle; sEV, small EV.

Considering the previous results, we hypothesised that the composition of EVs could be altered by infection, which would explain their differential effect on the phenotype of NSCs. Although our previous work has shown that EV protein composition is altered following infection, the predicted modified pathways pointed upon IPA analysis of EV protein composition were not clearly linked to neuronal differentiation (Bergamelli et al. 2022). This led us to investigate the miRNA content of placental EVs, given their pivotal role in neurodevelopment. We began our analysis by focusing on the miRNA content of sEVs derived from uninfected cells (Figure 5). A total of 448 miRNAs were detected in at least one of five replicates, with 164 miRNAs consistently present across all replicates. Notably, 10 miRNAs were particularly detected in placental sEVs (Figure 5A), including hsa‐let‐7a‐5p and hsa‐miR‐21‐5p, which are highly expressed in EVs across various biological contexts (EVAtlas, https://guolab.wchscu.cn/EVAtlas/#/rna) (Liu et al. 2022). Interestingly, let‐7 miRNA has been shown to play a role in the regulation of migration of newborn neurons (Petri et al. 2017).

We then conducted a direct target search for these miRNAs using the IPA ‘mRNA target filter tool’, focusing only on experimentally validated targets. A total of 462 validated targets of the 10 major miRNAs were identified and subjected to IPA core analysis. The ‘Top Diseases and Functions’ analysis highlighted roles in processes associated with the regulation of cell proliferation and migration (Figure 5B), critical during development. Among the ‘Top Canonical Pathways’ regulated by these miRNA targets, the ID1 signalling pathway – a CNS‐related pathway implicated in brain development – emerged (Fathi et al. 2011; Mizeracka et al. 2013; Liu et al. 2013) (Figure 5C). Furthermore, the ‘Upstream Pathways’ analysis revealed the involvement of key developmental regulators, including TGFβ (Meyers and Kessler 2017) and TP53 (Xiong et al. 2020; Hede et al. 2011) (Figure 5D).

**FIGURE 6:**
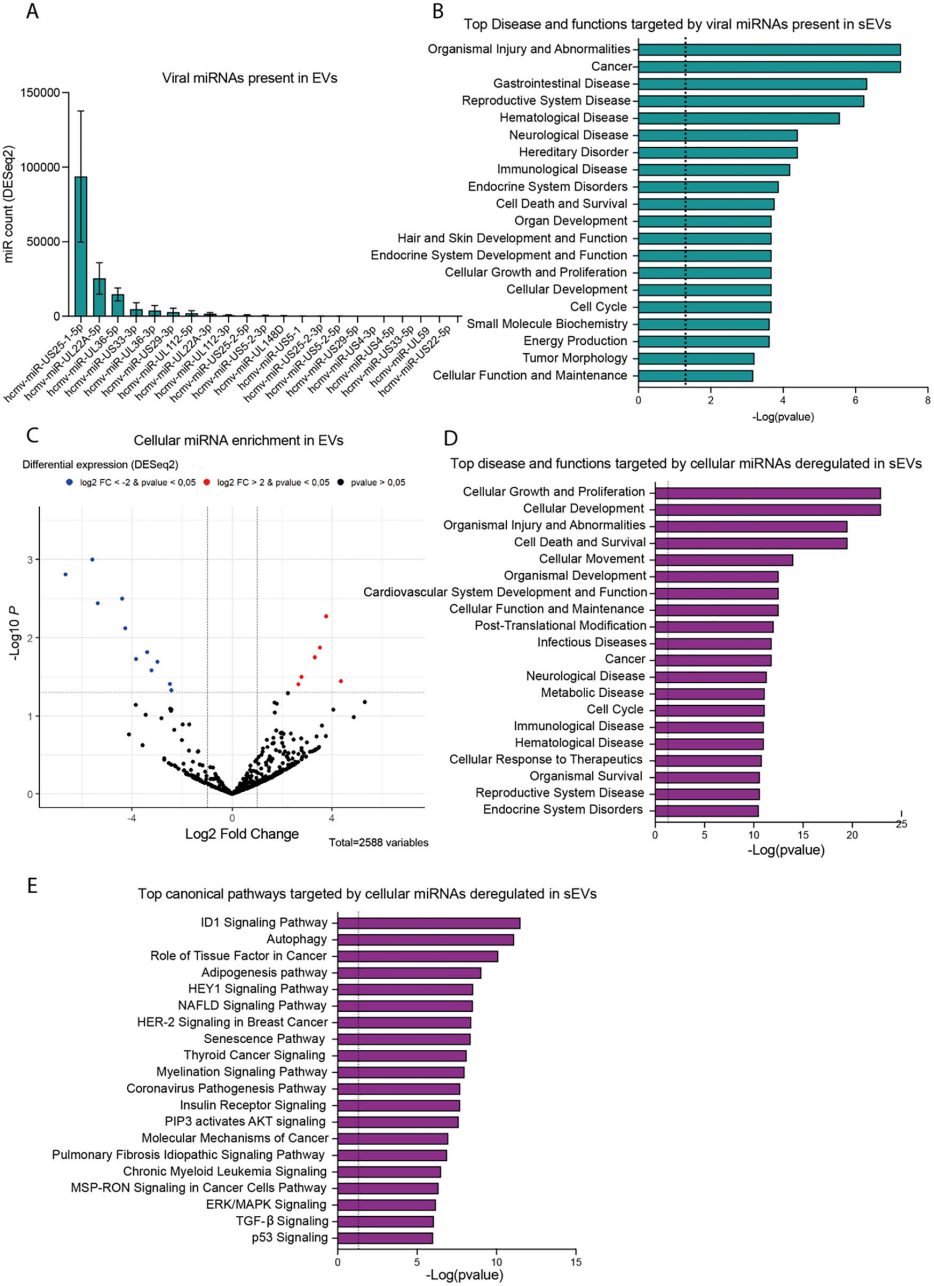
HCMV infection alters the miRNA landscape of placental small EVs. (A) Viral miRNAs present in sEVs prepared from placental cells. Histogram represents the mean ± SEM of miRNA counts found in sEVs upon normalisation (*N* = 5). (B) QIAGEN Ingenuity Pathway Analysis on the predicted cellular targets (predicted by miRDB) of the three highest expressed viral miRNAs found in placental sEVs. Histogram representing the Top 20 ‘Disease and functions’ categories. (C) Volcano‐plot representing the differential expression (DESeq2) of cellular miRNAs in sEVs upon infection by HCMV. Cellular miRNAs exhibiting significant differences between the HCMV versus uninfected conditions are represented by circles. Red: over‐represented miRNAs; blue: under‐represented miRNAs (*p* value ≤ 0.05 and log2 ratio ≥ 2 or ≤ −2, *N* = 5). (D) Top 20 ‘Disease and functions’ categories and (E) ‘Canonical pathways’ that may be impacted by the mRNA targets (‘experimentally proved’ in mRNA target filter) of the 18 differentially regulated miRNAs, ranged according to their *p* value. The dotted line represents the 0.05 *p* value threshold. EV, extracellular vesicle; HCMV, human cytomegalovirus; sEV, small EV.

We next compared the miRNA profile of placental sEVs produced by HCMV‐infected cells to that of uninfected cells (Figure 6). Like other herpesviruses, HCMV encodes a large repertoire of miRNAs that regulate both viral and host gene expression (Zhang et al. 2020). Of the 26 mature HCMV miRNAs identified to date, 21 were detected in sEVs derived from HCMV‐infected placental cells (Figure 6A and Table S6). Three miRNAs were particularly abundant in placental EVs: hcmv‐miR‐US25‐1‐5p, hcmv‐miR‐UL22A‐5p and hcmv‐miR‐UL36‐5p. Remarkably, hcmv‐miR‐US25‐1‐5p has been shown to interfere with neural progenitor cell fate by targeting Jag1 (Jiang et al. 2023). Given the limited information on HCMV miRNA targets, we conducted an in silico target prediction using the miRDB tool (Chen and Wang 2020) with a target score threshold of 50. A total of 316 predicted targets of the three major viral miRNAs were identified and subjected to IPA core analysis (Figure 6B). The ‘Top Diseases and Functions’ analysis for these targets revealed processes related to development, including ‘Cell death and survival’, ‘Organ development’ and ‘Hair and skin development and function’. Pathology‐related categories such as ‘Organismal injuries and abnormalities’, ‘Neurological diseases’ and ‘Immunological diseases’ were also prominent.

We further analysed the cellular miRNAs differentially expressed in placental sEVs during HCMV infection. A total of 18 cellular miRNAs exhibited significant alterations in abundance upon infection, with 7 miRNAs upregulated and 11 downregulated, notably hsa‐miR‐125, playing a role in neuronal commitment (Boissart et al. 2012) (Figure 6C and Table S7). An IPA core analysis of the 74 experimentally validated targets for these miRNAs revealed significant associations with development‐related processes, including ‘Cellular growth and proliferation’, ‘Cellular development’ and ‘Cell death and survival’ (Figure 6D). Pathology‐related terms such as ‘Organismal injuries and abnormalities’, ‘Neurological diseases’ and ‘Infectious diseases’ were also identified. Interestingly, several ‘Top Canonical Pathways’ significantly affected upon HCMV infection were linked to development, particularly brain development. These included the ID1 signalling pathway (Fathi et al. 2011; Mizeracka et al. 2013; Liu et al. 2013), autophagy (Nagayach and Wang 2024), HEY1 signalling pathway (Lasky and Wu 2005), and pathways involving TGFβ1 (Meyers and Kessler 2017) and TP53 (Xiong et al. 2020; Hede et al. 2011) (Figure 6E).

Of note, an RNase protection assay was carried out both on EV preparations from uninfected or HCMV‐infected cells. Even if a fraction of miRNAs were partially degraded upon RNase treatment alone (similar to detergent treatment alone), a pretreatment of EVs by detergent followed by incubation with RNase led to the significant degradation of a large proportion of miRNAs, indicating that the majority of cellular and viral miRNAs were contained in EVs (Supporting Information and Figure S7).

### 3.6 Integrative Analyses Link Dysregulated Placental EVs‐Derived miRNAs to the Regulation of Their Predicted Target Genes

**FIGURE 7:**
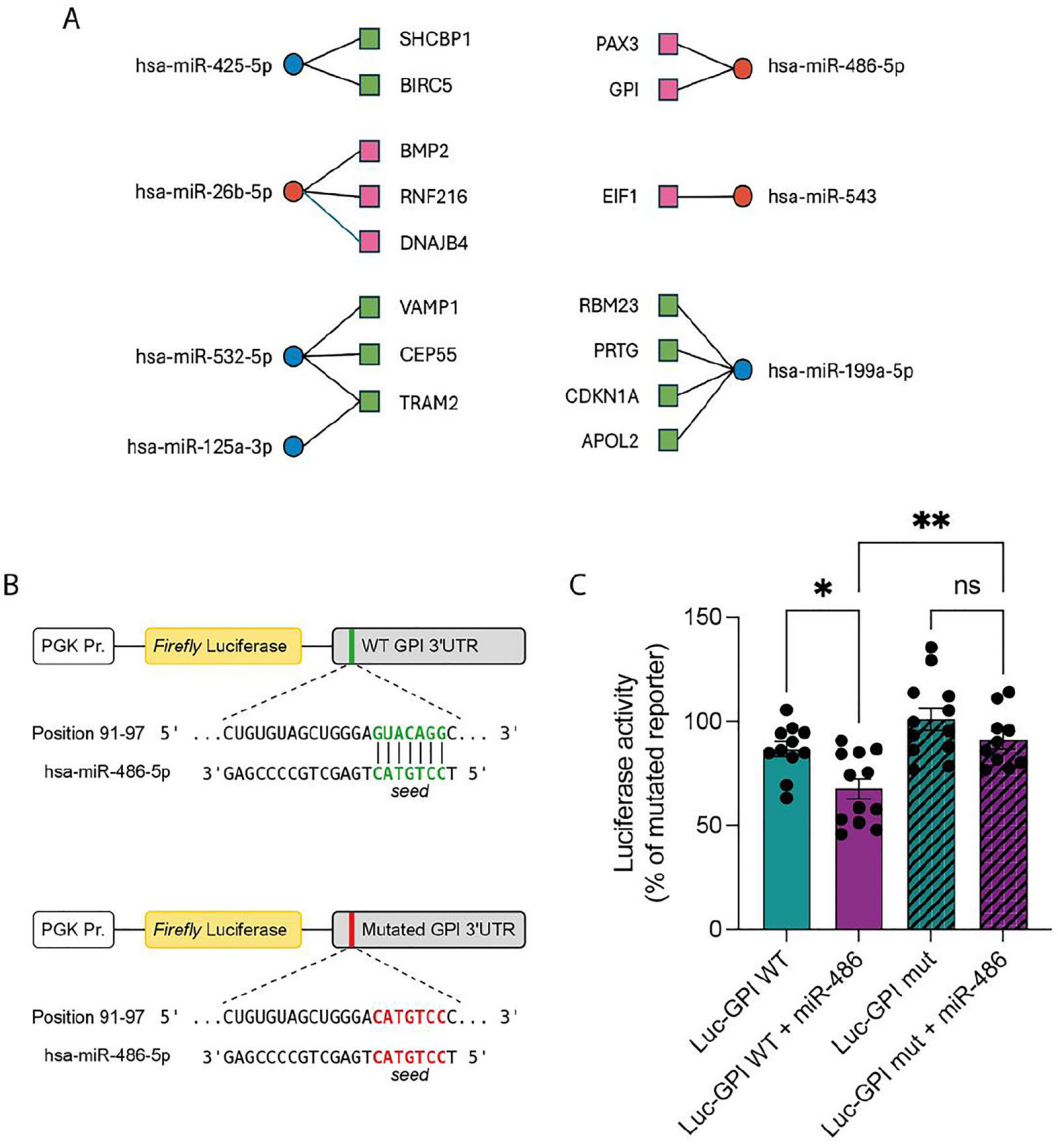
miRNAs dysregulated in placental EVs can modulate the expression of predicted target genes identified in NSCs. (A) Integrated network of dysregulated EV‐miRNAs and inversely regulated target genes in recipient NSCs. The panel illustrates selected miRNA–target pairs showing inverse expression patterns between EV‐derived miRNAs (from HCMV‐infected placental cells) and their putative target mRNAs or proteins identified in recipient NSCs from the RT^2^ and proteomic datasets. Only interactions supported by experimentally validated (miRTarBase) or high‐confidence predicted (TargetScan) evidence, and consistent with miRNA‐mediated repression (miRNA up/down vs. target down/up), are shown. Each miRNA (circle) is connected to its corresponding target gene (square), with colour indicating the direction of regulation (red = upregulated miRNAs; blue = downregulated miRNAs; green = upregulated targets; pink = downregulated targets). (B) Schematic representation of the constructs used in the luciferase reporter assay. The GPI 3′UTR (wild type, WT; or mutated, mut) was cloned downstream of the *Firefly* luciferase reporter gene. The alignment between the WT GPI 3′UTR complementary sequence and the seed region of has‐miR‐486‐5p is shown. In the mutated construct, the seed‐matching sequence was replaced by its complementary sequence, thereby preventing miR‐486‐5p binding. (C) Functional validation of the GPI 3′UTR as a direct target of miR‐486‐5p. HEK‐293 cells were cotransfected with the luciferase reporter plasmid containing the GPI 3′UTR (WT or mut) and either a miR‐486‐5p mimic or control vector. Luciferase activity was normalised to the value obtained with the mutated reporter construct (set as 100%). Data represent mean ± SEM from at least 10 independent experiments (represented by individual dots for each bar). A one‐way ANOVA test indicates a *p* value <0.0001 between the different conditions. A Tukey post‐hoc test was realised, which results are indicated above the histogram (*p value < 0.05; **p value < 0.01). EV, extracellular vesicle; HCMV, human cytomegalovirus; NSC, neural stem cell; sEV, small EV.

Finally, to further explore the potential impact of the dysregulated miRNAs on the gene expression program of recipient NSCs, an integrated analysis combining the miRNA, RT^2^ and proteomic datasets was performed (Figure 7A and Table S8). Notably, 16 miRNA–target pairs exhibited inverse expression patterns consistent with miRNA‐mediated repression (e.g., upregulated EV‐miRNAs associated with downregulated target genes or proteins). Thus, we examined whether one of the identified dysregulated miRNAs (hsa‐miR‐486‐5p, upregulated in EVs upon infection) may directly target the expression of one of its putative target genes (GPI, downregulated in NSCs upon incubation with HCMV EVs).

To directly assess the ability of miR‐486 to repress GPI translation through binding to its 3′UTR, we generated reporter plasmids by fusing the 3′UTR region of GPI containing the hsa‐miR‐486‐5p binding‐site to the 3′ terminus of the firefly luciferase coding sequence (Figure 7B and Tables S3 and S4). We then coexpressed this construct in HEK‐293 cells together with hsa‐miR‐486‐5p and measured luciferase activity (Figure 7C). hsa‐miR‐486‐5p expression significantly decreased the luciferase signal by around 22% compared to the control condition where hsa‐miR‐486‐5p was not overexpressed. Importantly, this effect was prevented when the binding site for hsa‐miR‐486‐5p was mutated in the luciferase reporter, confirming that hsa‐miR‐486‐5p can inhibit translation of GPI by directly interacting with its 3′UTR.

Collectively, these results highlight candidate miRNA–target interactions that may underlie the transcriptomic and proteomic remodelling observed in recipient NSCs. Together, they provide a coherent integrative view supporting the contribution of EV‐associated miRNAs to the modulation of neurogenic pathways upon HCMV infection.

In conclusion, our data reveal that HCMV infection profoundly reshapes the miRNA landscape of placental sEVs, with a notable enrichment in neurodevelopment‐related pathways. These findings suggest that placental sEV miRNAs may play a key role in regulating the NSC phenotype, an effect observed with sEVs from noninfected cells but disrupted when derived from HCMV‐infected cells. This highlights a potential mechanistic link between altered sEV miRNA cargo and the neurodevelopmental impact of HCMV on the foetal brain.

## 4 Discussion

In recent years, a growing number of studies have highlighted the pivotal role of placental EVs throughout pregnancy. Most studies to date have focused on their roles in placental establishment, embryo implantation and maternal immune tolerance, as well as their dysregulation in pregnancy pathologies such as diabetes mellitus and preeclampsia (Martin et al. 2022). However, despite placental EVs account for almost half of the EVs circulating in foetal blood (Miranda et al. 2018), little is known about their contribution to foetal development, particularly in the foetal brain. In this study, we investigated whether the phenotype of NSCs is influenced by placental sEVs, and whether it can be impaired upon HCMV infection of placental cells.

To examine these questions, we used the HIPEC model, derived from primary first‐trimester extravillous cytotrophoblasts (Pavan et al. 2003). This model, used in other studies focusing on HCMV placental infection (Rauwel et al. 2010; Leghmar et al. 2015; Njue et al. 2023), was selected because it can be expanded to yield sufficient EVs and is permissive to HCMV infection without overt cell death before sEV preparation, which is critical for downstream analyses. However, we are fully aware that this cell line represents only one facet of trophoblast biology. Extravillous cytotrophoblasts are mainly specialised for decidual invasion and spiral artery remodelling, and other trophoblast populations, such as the syncytiotrophoblasts, contribute significantly to the placental EV repertoire. Thus, our findings must be interpreted with the caution that they do not capture the full complexity of placental EVs. Nonetheless, our previous results demonstrating the proviral activity of EVs isolated from primary human placental histocultures could also be observed using HIPEC‐derived EVs (Bergamelli et al. 2022). This supports the relevance of the model as a reductionist, yet informative, system for dissecting the molecular cargo and functional roles of placental EVs on neural precursors.

During proper foetal brain development, NSCs first proliferate before migrating to their final destination, where, once their bona fide positioning is achieved, they subsequently differentiate into neurons or glial cells (Guerrini and Parrini 2010). Interestingly, under noninfected conditions, we observed that placental EVs exerted a significant effect on NSC differentiation and migration compared to control‐treated NSCs. Placental sEVs from uninfected cytotrophoblasts appeared to promote neurogenesis by enhancing the expression of genes and proteins involved in neural cell fate and neuronal homeostasis, while reducing the migration capacities of NSCs. These effects suggest that placental EVs could participate to the orchestration of the different steps of neurogenesis in physiological conditions. It should be noted, however, that the NSCs used in these studies primarily give rise to glutamatergic neurons, and that the impact of placental EVs on neuronal differentiation and migration may differ across neuronal subtypes.

Strikingly, the effect of placental sEVs on neurogenesis was abolished when NSCs were incubated with placental sEVs derived from HCMV‐infected cytotrophoblasts, with an increase in their migration celerity correlated with a reduced differentiation toward neurons – and expression of lineage‐specific genes – which return to the baseline observed for untreated NSCs. sEVs secreted by the infected placenta make neural progenitors more prone to migration compared to sEVs from uninfected placenta, which could contribute to migration abnormalities, such as polymicrogyria, observed in congenital HCMV infection (Leruez‐Ville et al. 2020). Additionally, this may hinder their ability to differentiate, which was already compromised after incubation with sEVs from HCMV‐infected placental cells. Thus, during HCMV congenital infection, sEVs from infected placenta seem to lose the neurogenic potential observed for sEVs from uninfected placenta. This emphasises the importance of placental health and integrity for foetal neurodevelopment, and its compromise following a viral attack on the placenta.

To further explore the mechanism underlying these detrimental effects, we analysed the molecular composition of placental sEVs. Our prior research demonstrated that HCMV infection alters the protein content of placental sEVs, increasing NSC permissiveness to viral infection (Bergamelli et al. 2022; Bergamelli et al. 2021). Here, we expanded this characterisation by analysing the miRNA cargo of placental sEVs and found significant alterations associated with HCMV infection. Notably, sEVs from HCMV‐infected cytotrophoblasts incorporated viral miRNAs described as a critical regulator of neurogenesis (Jiang et al. 2023). Additionally, we identified 18 cellular miRNAs whose expression levels were altered by infection. IPA functional analysis of their experimentally validated targets highlighted developmental pathways involving ID1, HEY1 and TGFβ, all of which are interrelated (Zhang et al. 2021; Liu et al. 2007; Sivertsen et al. 2007), and play key roles in neurogenesis (Aloia et al. 2015; Zhang et al. 2020; Harada et al. 2021; Than‐Trong et al. 2018; Watanabe et al. 2022). Our integrated analysis combining miRNA, transcriptomic and proteomic data provided additional support for the involvement of placental EV miRNAs in regulating NSC gene expression. Several dysregulated miRNAs exhibited inverse expression patterns with their predicted or validated targets in recipient NSCs, consistent with miRNA‐mediated repression. Additionally, we demonstrated that one of the putative identified target genes, GPI, was regulated by the exogenous expression of hsa‐miRNA‐486‐5p, overexpressed in placental EVs upon infection. Although we have not formally proven direct miRNA–target interactions for all the predicted targets in this study, the convergence of multi‐omic evidence supports a model whereby HCMV‐induced alterations in EV‐miRNA cargo contribute to the dysregulation of neurogenic pathways in recipient cells. These findings are in line with previous reports describing EV‐mediated modulation of recipient cell transcriptomes via transferred miRNAs (Kambe et al. 2014; Li et al. 2020) and further highlight the potential regulatory impact of placental EV cargo on foetal neural development.

The enrichment of 21 out of 24 HCMV‐derived miRNAs in EVs secreted by infected HIPECs highlights a further level of the interplay between viral replication and host EV biogenesis (Martin et al. 2023; Nolte‐’t Hoen et al. 2016). Previous studies including ours have shown that herpesviruses and in particular HCMV exploit the exosomal pathway for virion maturation and egress, largely through shared interaction with the ESCRT machinery and multivesicular body trafficking (Bergamelli et al. 2022; Turner et al. 2020; Zicari et al. 2018; Sadeghipour and Mathias 2017). Our current findings extend this concept by showing that HCMV selectively packages viral miRNAs into placental EVs, in addition to the previously described incorporation of viral proteins. This confirms the coevolutionary strategy whereby viral materials are disseminated via EVs, potentially modulating recipient cell responses and contributing to viral dissemination and pathogenesis.

The functional role of miRNAs within EVs remains highly debated in the scientific community, particularly due to the low abundance of miRNAs in EVs and their limited delivery to recipient cells, raising questions about the extent of their biological effects (Jeppesen et al. 2019; Makarova et al. 2021; Albanese et al. 2021). However, it is crucial to consider the high abundance of EVs in the bloodstream, approximately 10^10^ EVs/mL, which continuously interact with recipient cells (Auber and Svenningsen 2022), and that nearly 50% of them are of placental origin in the developing foetus (Miranda et al. 2018). Some studies suggest that EV‐mediated transfer of placental miRNAs and subsequent modulation of their target genes occurs in recipient cells, including in vitro in Jurkat cells and in vivo in maternal NK cells (Kambe et al. 2014). Another study demonstrated EV‐mediated internalisation of placental miRNAs in neighbouring primary placental and uterine cells, where they retained biological activity. This study also reported an association between EV miRNAs and P‐body proteins such as AGO2, supporting the functionality of EV‐transferred miRNAs (Li et al. 2020). In our current study, we ensured the use of physiological EV doses, by incubating NSCs with approximately 1000 EV/cell, consistent with our recovery yield around 2000 EVs/cell using a highly pure but low‐yield gold‐standard preparation method combining differential and density gradient ultracentrifugation. Although IPA analyses pointed to dysregulation of neurogenesis pathways by placental miRNAs, and we observed functional impacts of placental sEVs – whether derived from uninfected or HCMV‐infected cytotrophoblasts – on NSC phenotype, we cannot exclude that miRNAs are not the only mediators of EV effects. Notably, the protein composition of placental sEVs is also altered upon HCMV infection, with a positive activation for the term ‘Formation of skin’, linked to the neuroectodermal layer and neural cells (Bergamelli et al. 2022). Thus, both protein and miRNA content of placental EVs could modulate NSC phenotype and neurogenesis, warranting a comprehensive mechanistic study to identify the specific components responsible for these effects.

Finally, direct HCMV infection of the foetal brain, particularly of NSCs, is well‐documented as a disruptor of brain development per se, through various mechanisms (Rolland et al. 2016; Brown et al. 2019; Li et al. 2024; Niu et al. 2023; Wu et al. 2018). In a previous work, we demonstrated that placental EVs present a proviral function that favours HCMV dissemination in NSCs, potentiating the detrimental effect of HCMV infection on brain development (Bergamelli et al. 2022). Here, our data provide an additional layer to the understanding of pathophysiology in congenital infection, suggesting a complementary role of placental EVs, which may exert early deleterious effects – by themselves – even before viral dissemination within the foetal brain.

**FIGURE 8:**
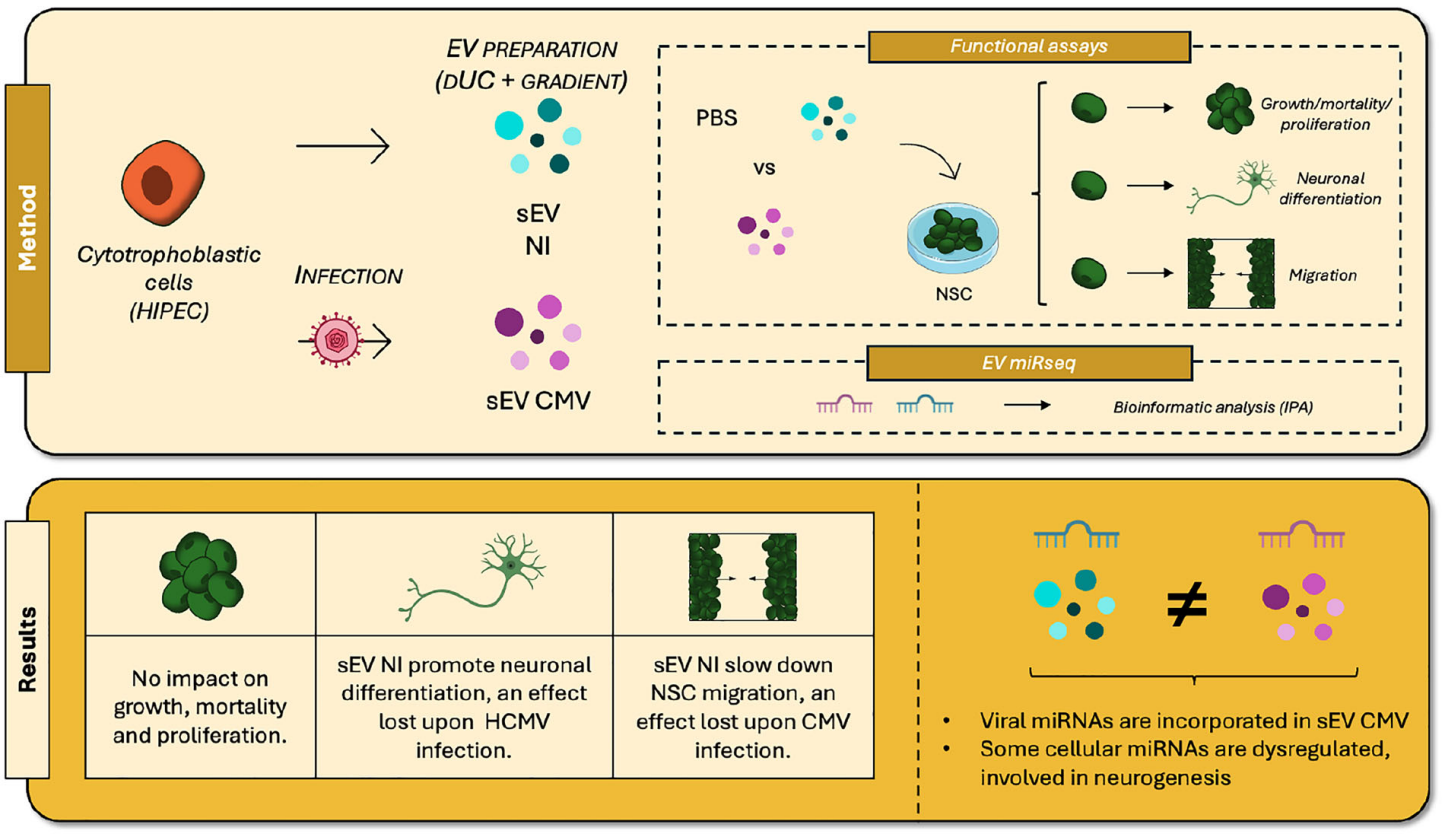
Graphical summary of the study.

In conclusion, our study highlights the role of placental EVs in foetal brain development, promoting neurogenesis under physiological conditions and compromising it under conditions of HCMV infection (Figure 8). These results reveal the importance of placental EV‐mediated communication in shaping foetal brain development, a relatively unexplored area (Gall et al. 2022, Gumusoglu 2024). Investigating the role of placental EVs in neurogenesis during both normal and pathological pregnancies may provide valuable insights into pregnancy complications. Moreover, the altered miRNA composition of placental EVs during HCMV infection underscores their potential as non‐invasive biomarkers for pathological pregnancies. Although severe brain abnormalities can be detectable via ultrasound, milder lesions, such as calcifications, often go unnoticed, and neurosensory impairments remain undetectable. Future studies will be needed to formally demonstrate the mechanistic link between changes observed in the miRNA profiles of EVs and neural development of the foetus. In any event, our study provides a strong incentive to gain further insight on the potential roles of EVs in normal or pathological pregnancies, as it may offer new opportunities for diagnosing or managing affected mother and child.

## Funding

This study was funded by the INSERM, CNRS, Toulouse University, the Biomedicine Agency (grants 19AMP008; 24AMP009) and the Réseau Mère Enfant de la Francophonie (RMEF). Charlène Martin's thesis was funded by the French Ministry of Education and Research (MESR), and her work is supported by the Villa M foundation.

## Conflicts of Interest

The authors declare no conflicts of interest.

## Supporting information



Supplementary data

## Data Availability

The data that support the findings of this study are openly available in PRIDE, reference number PXD060921 for proteomic data, SRA database (NCBI) reference PRJNA1129057 for miRNA sequencing data and at Github (https://github.com/CMart217/R‐script—differential‐expression‐of‐miRNA) for miRNA analysis.
