# Supplementary material for: Placental Extracellular Vesicles Exhibit Reduced Neurogenic Potential Linked to Changes in Their miRNA Landscape Upon HCMV Infection
Source: J Extracell Biol. 2026 Jan 25;5(1):e70108. doi: 10.1002/jex2.70108 (PMC12832074; doi:10.1002/jex2.70108)
Supplement: Supplementary file 1 — Supplementary data [file JEX2-5-e70108-s001.docx]

Supplementary data

# Materials and methods

**RNase protection assay.**

EV preparations (uninfected or HCMV-infected) were equally divided into four equal volume fractions, adjusted to 100 μL each. Fractions were then treated with 1.25 μL of 0.1% Triton-X100 (ThermoFischer Scientific, or PBS for the untreated control) during 30 min at 4 °C followed by the addition of 2.5 μL of RNase A/T1 treatment (ThermoFisher Scientific, or PBS for the untreated control) during 15 min at 37 °C. RNAs were then extracted by using the Direct-zol RNA Microprep Kit (Zymo Research), following the manufacturer’s instructions. 2 μL out of 11 μL total extracted RNAs were then subjected to reverse transcription, using miRCURY LNA RT Kit (Qiagen), which contains an UniSP6 spike-in RNA for normalization of the downstream qPCR reactions. For each condition, the qPCR quantification of three miRNAs (two cellular miRNAs, hsa-miR-27b-3p and hsa-miR30d-5p, found invariant in our miRNA-seq dataset and one viral hcmv-miR-US25-1p) as well as the UniSP6 spike-in, was then performed using Qiagen miRCURY LNA SYBR^®^ Green miRNA PCR assay, with commercially designed probes. Quantification of the miRNA expression was done by the ΔΔCt method using UniSP6 as reference miRNA and the untreated condition as the normalization condition.

## Supplementary Table S1. Antibodies used for immunofluorescence and western-blot analyses.

| Name of the targeted protein | Dilution used | Reference |
| --- | --- | --- |
| Immediate Early (mouse) | 1/500 | IE1/IE2 CH160 ab53495, Abcam |
| SOX2 (rabbit) | 1/500 | Cell Signaling D6D9 #3579 |
| Nestin (mouse) | 4 µg/mL | Abcam 10C2 ab22035 |
| HUC/D (mouse) | 1/500 | 16A11, Life Technologies |
| GFAP (chicken) | 1/500 | #173006, Synaptic Systems |
| Ki67+ (rabbit) | 1/100 | KiS5 Millipore |
| DAPI | 1 µg/mL | Sigma |
| Goat anti-rabbit IgG (H+L)-Alexa fluor 594 | 1/1,000 | Life Technologies |
| Goat anti-mouse IgG (H+L)-Alexa fluor 488 | 1/1,000 | Life Technologies |
| Goat anti-chicken IgG (H+L)-Alexa fluor 488 | 1/1,000 | A11039 Invitrogen |
| CK7 (rabbit) | 1/5,000 | Genetex GTX109723 |
| Vimentin (mouse) | 1/1,000 | Santa Cruz sc-6260 |
| CD81 (mouse) | 1/1,000 | Santa cruz sc-166029 |
| CD9 (mouse) | 1/1,000 | Millipore CBL162 |
| Tsg101 (rabbit) | 1/1,000 | Abcam ab30871 |
| Alix (rabbit) | 1/1,000 | Abcam ab88388 |
| Tom20 (rabbit) | 1/1,000 | Abcam ab186735 |
| IRDye 680RD goat anti mouse | 1/10,000 | Licor 926-68070 |
| IRDye 800CW goat anti rabbit | 1/10,000 | Licor 926-32211 |

## Supplementary Table S2. Primers.

| Gene of interest | Primer sense | Primer sequence (5’ to 3’) |
| --- | --- | --- |
| *sox2* | Forward | TCGGCATCGCGGTTTTT |
|  | Reverse | ACAGCAAATGACAGCTGCAAA |
| *nestin* | Forward | GGAAGAGAACCTGGGAAAGG |
|  | Reverse | CTTGGTCCTTCTCCACCGTA |
| *hucd* | Forward | TGCTACGGAACCGATTACTGT |
|  | Reverse | TGGTCCAGACATCAGTCTCTTT |
| *map2* | Forward | AAAGCTGATGAGGGCAAGAA |
|  | Reverse | GGCCCCTGAATAAATTCCAT |
| *gfap* | Forward | AGAAGCTCCAGGATGAAACC |
|  | Reverse | AGCGACTCAATCTTCCTCTC |
| *gapdh* | Forward | GAAGGTGAAGGTCGGAGTC |
|  | Reverse | GAAGATGGTGATGGGATTTC |
| *hprt* | Forward | CGAGATGTGATGAAGGAGATGG |
|  | Reverse | TGATGTAATCCAGCAGGTCAGC |

## Supplementary Table S3. Details of the miRNA used in the luciferase reporter assay

| **miR** | **Seed (5'-3') miRNA** | **Target** | **Region in 3'UTR** |
| --- | --- | --- | --- |
| hsa-miR-486-5p | CCUGUAC | GPI | 91-97 |

## Supplementary Table S4. Plasmids used and constructed for the luciferase reporter assay

|  |  |  |
| --- | --- | --- |
| **Plasmids** | **Relevant characteristics** | **Sources** |
| pcDNA3.1(+) | Amp^r^, Hyg^r^, CMV promoter, SV40 promoter, ColE1 origin | Thermo-Fisher |
| pmiR_486 | pcDNA3.1(+) derivative with production of miRNA-486 | This study |
| pmiRGLO | Amp^r^, Kan^r^, Neo^r^, PGK promoter, SV40 promoter, ColE1 origin, Firefly luciferase (luc2), Renilla luciferase (hRluc-neo) | Promega |
| pmiRGLO-GPI WT | pmiRGLO derivative with miRNA-486 target site for GPI gene | This study |
| pmiRGLO-GPI mut | pmiRGLO-GPI derivative with mutated seed of the target site | This study |
|  |  |  |

Amp^r^: Ampicillin resistant; Kan^r^: Kanamycin; Neo^r^: Neomycin.

# Results

## Supplementary Figure S1. Characterization of the HIPEC model.


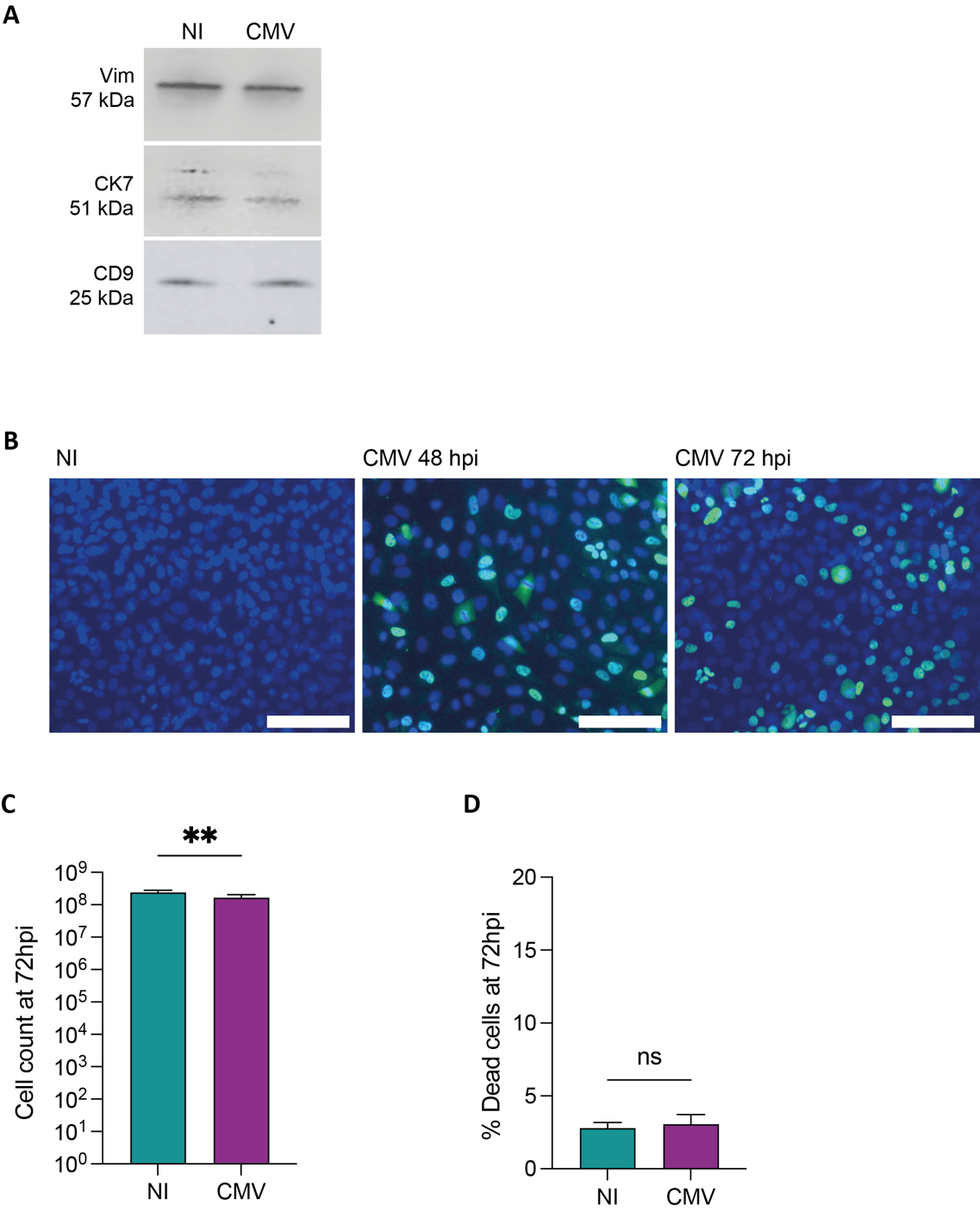


HIPECs were infected (CMV) or not (NI) by HCMV at an MOI of 10 during 72h. (A) Cells were subjected to western-blot analysis with the indicated antibodies (Vim: vimentin; CK7: cytokeratin 7). (B) The level of infection was assessed by immunofluorescence against IE antigen (IE: green; DAPI: blue) at different hours post-infection (hpi). Scale bar: 100 μm. Data presented in (A) and (B) are representative for three independent experiments. (C) Cell growth and (D) viability were assessed by counting the cells at 72 hpi with trypan blue. Histograms represent the mean ± SEM of at least 10 independent experiments. **: p<0.01 by paired t-test; ns: non-significant.

## Supplementary Figure S2. Study of PKH67-labelled placental EVs captured by NSCs by flow cytometry.

**
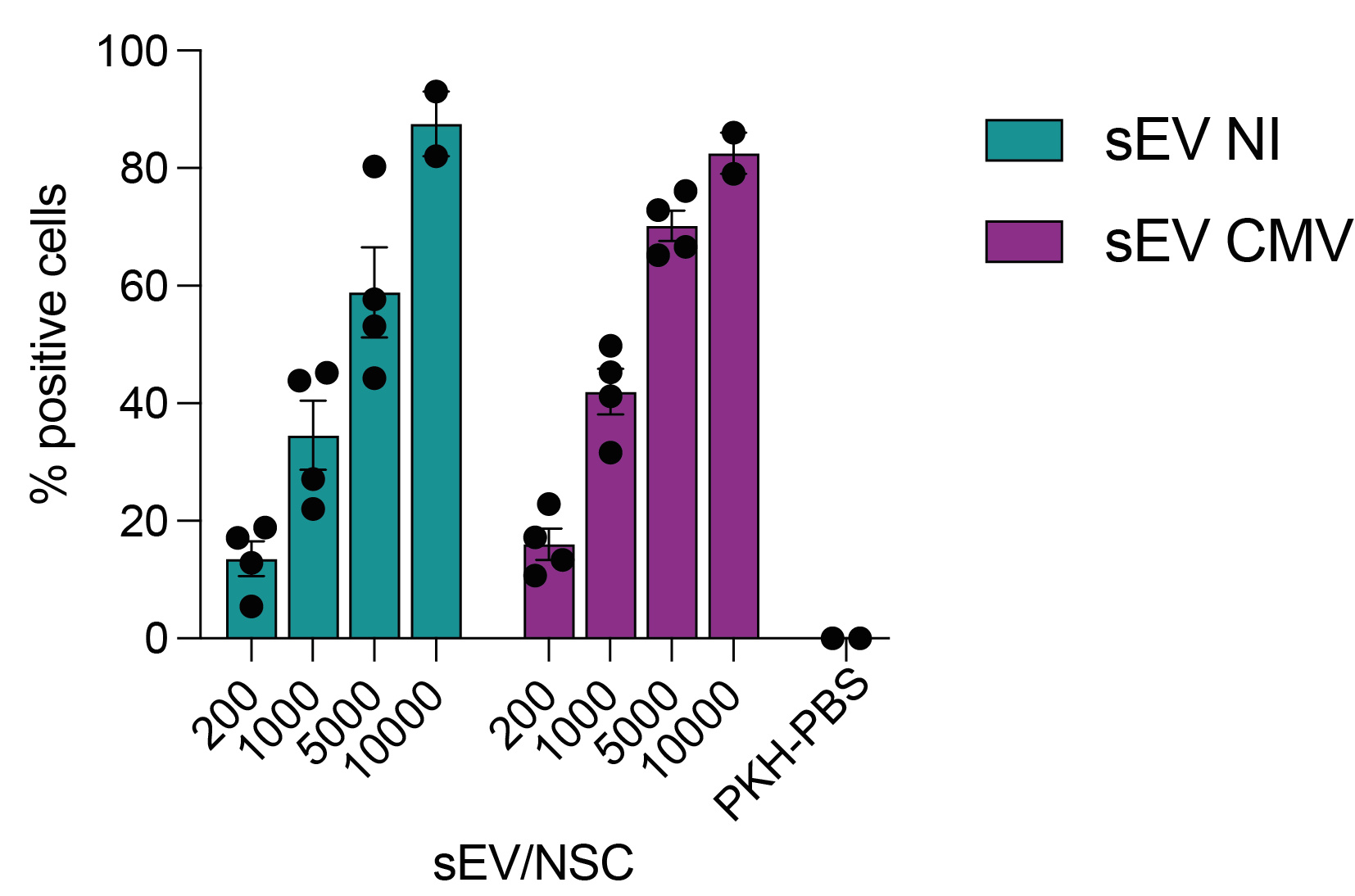
**

NSCs were seeded at day 0 (D0) and four different amounts of PKH67-labelled sEVs (200; 1,000; 5,000 or 10,000 sEV/cell) or PKH67-stained PBS (PKH-PBS) were added at D1. The PKH-PBS control was realized to rule out any potential uptake of dye micelles by cells (with a volume equivalent to a dose of 10,000 sEV/cell). Measurements of fluorescent positive NSCs was performed 24h later by flow cytometry. All results are represented as mean ± SEM (N=2 to 4). A two-way ANOVA test was performed. A significant effect of time was observed (p<0.0001) but no significant difference was observed depending on the HCMV status of sEVs.

## Supplementary Figure S3. Impact of increasing concentrations of placental EVs on the growth, mortality and proliferation of NSCs.

**
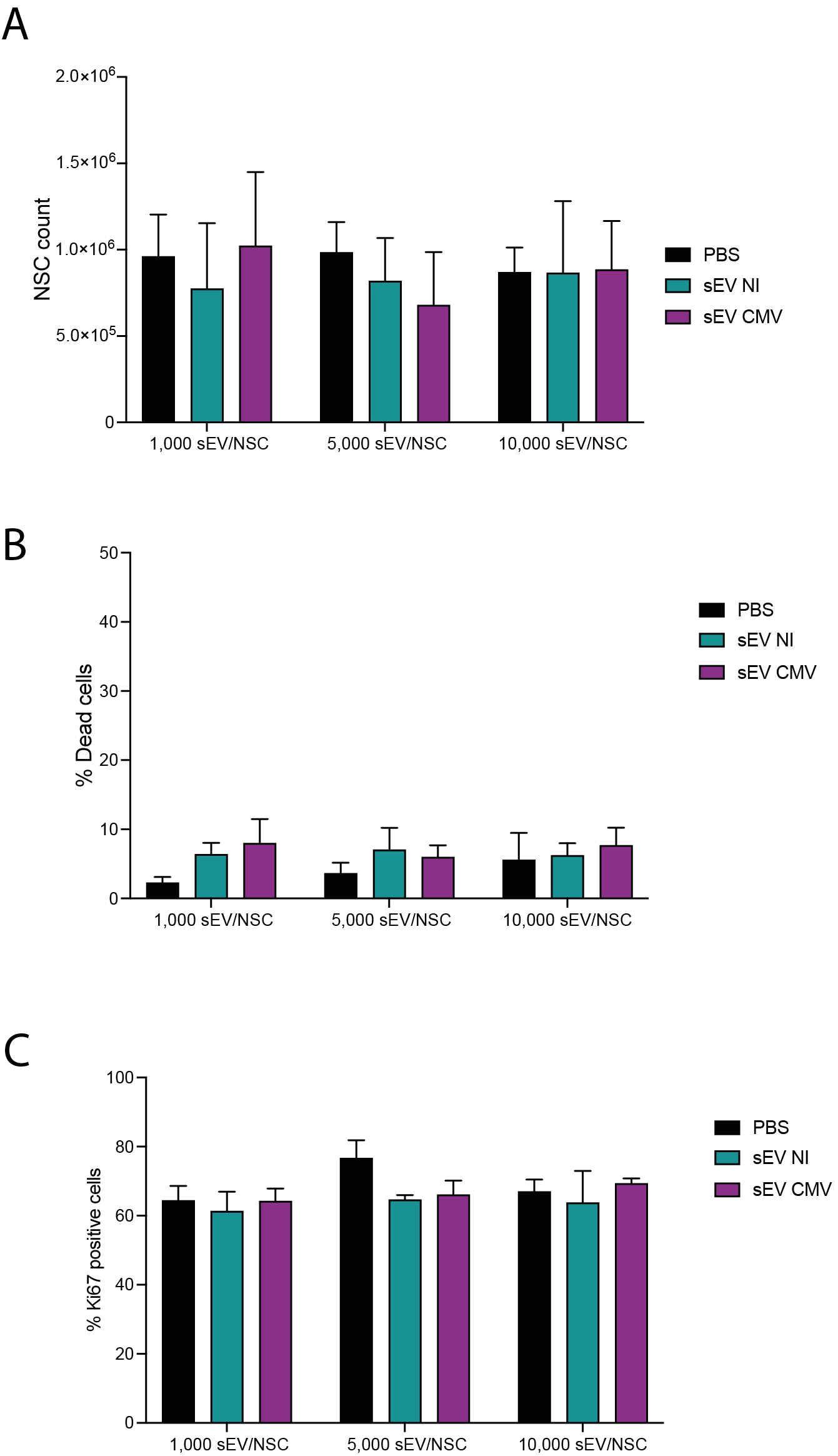
**

Cells were seeded at day 0 (D0) and three different amounts of sEVs (1,000; 5,000 or 10,000 sEV/cell) were added at D1. Measurements of the different parameters were performed at D4 as in Figure 1. (A) The number of NSCs was determined by counting, N=3. (B) The mortality of NSCs was evaluated by trypan blue exclusion, N=3. (C) The proliferation of NSCs was evaluated by performing an anti-Ki67 immunofluorescence, N=3. All results are represented as mean ± SEM. For each experiment, a two-way ANOVA test was performed and was not significant.

## Supplementary Figure S4. NSC differentiation determined by RT-qPCR on cell type-specific genes.

**
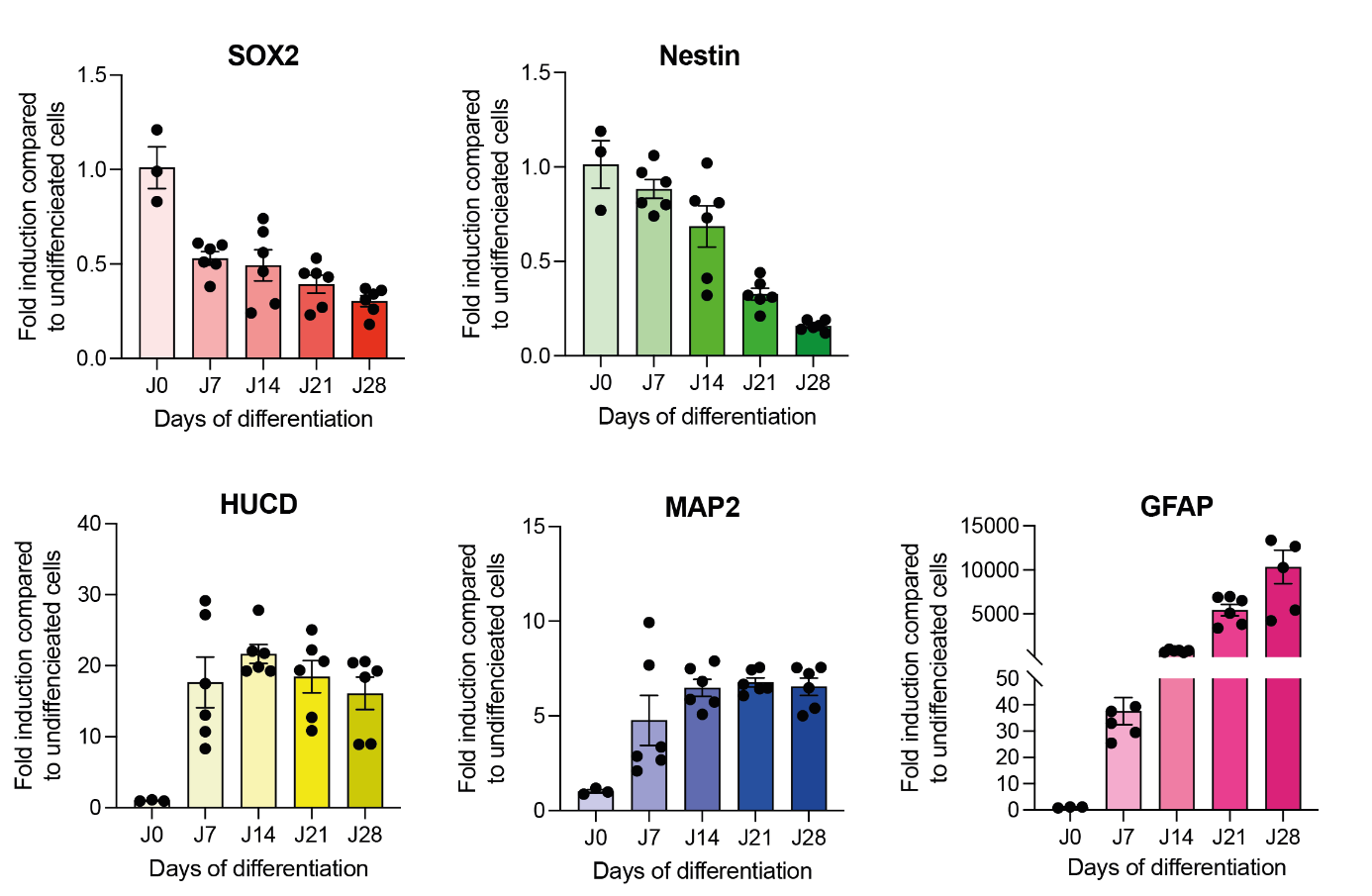
**

NSCs were seeded at day 0 in the presence of BDNF to trigger differentiation. At different times post differentiation, RNA was extracted and subjected to qRT-PCR to follow the expression of genes indicated on the top of the histograms, using the day 0 as the normalization condition. All results are represented as mean ± SEM. N=6.

## Supplementary Figure S5. RT2 profiler analysis of genes induced upon NSCs differentiation.

**
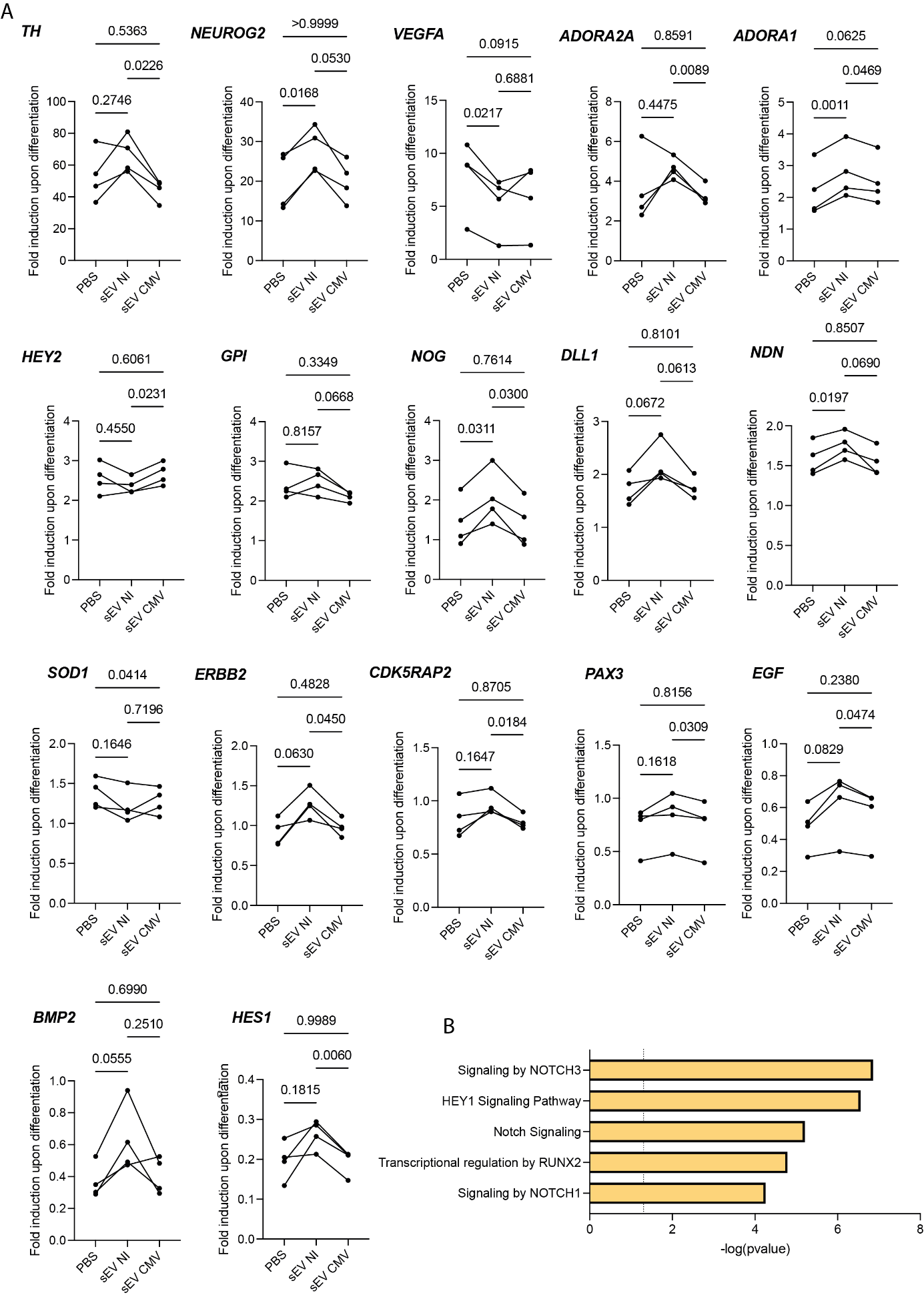
**

(A) Graphs representing the expression of the 17 individual genes identified as significantly differentially induced upon differentiation, depending on the treatment by placental sEVs, during the RT^2^-profiler PCR array “Neurogenesis” analysis. Each point/line represents an independent replicate. Above the graphs are indicated the adjusted *p*-values calculated upon the Tukey’s post-hoc statistical test realized after the one-way ANOVA. N=4. (B) IPA analysis indicating the 5 top upstream regulators of the 17 previous identified genes.

## Supplementary Figure S6. Proteomic analysis of NSCs upon differentiation.


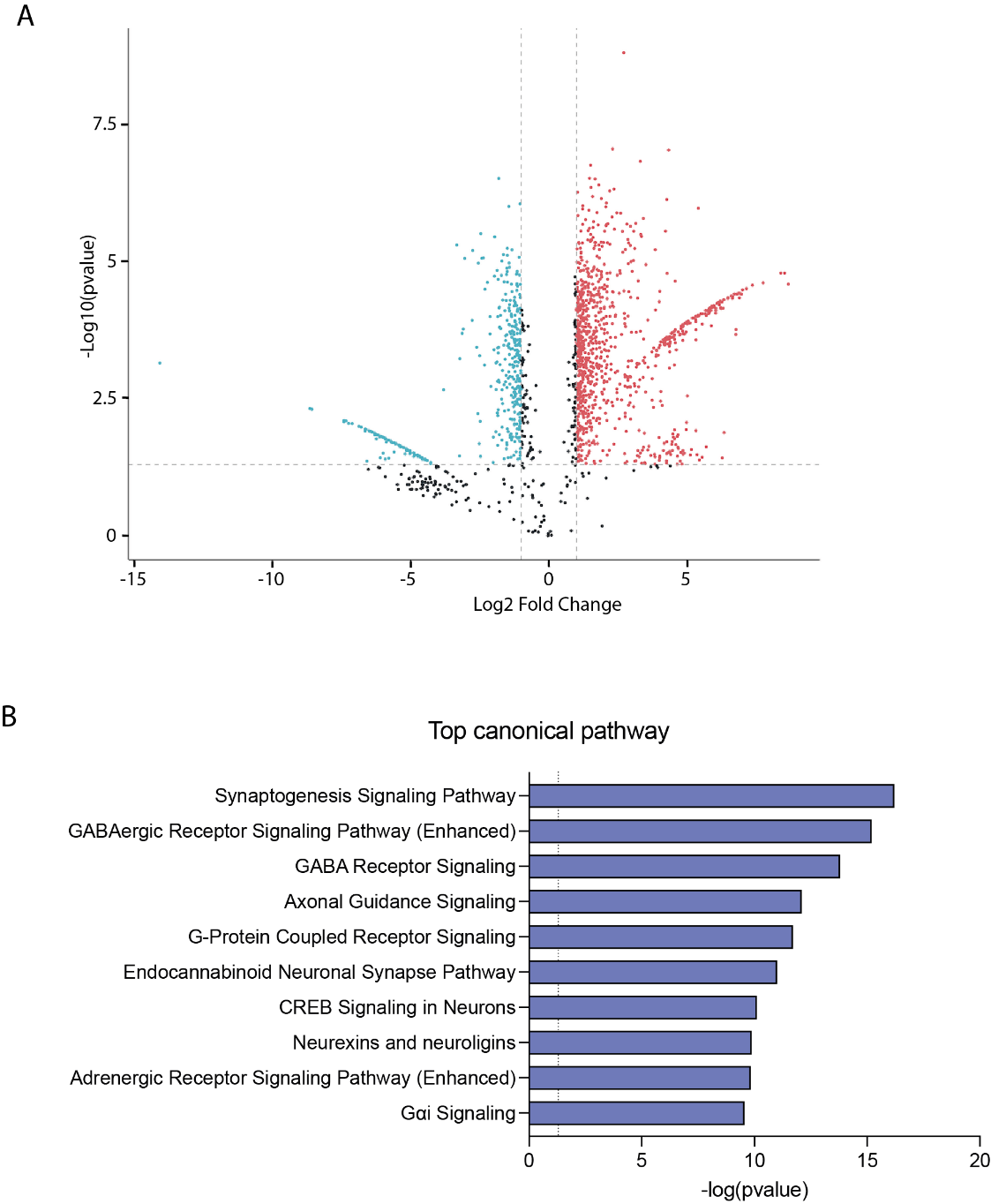


(A) Volcano-plot representing differences in normalized mean protein abundance in NSCs upon differentiation *vs* non-differentiated NSCs. Proteins exhibiting significant differences between the two conditions are represented by circles. Red: over-represented proteins; Blue: under-represented proteins (*p*-value ≤ 0.05 and log2 fold change ≥ 1 or ≤ −1; N=3). (B) IPA realized on the differentially expressed proteins. Histogram representing the top ten “Canonical pathways” categories, ranged according to their *p*-value. The dotted line represents the 0.05 *p*-value threshold.

## Supplementary Figure S7. Viral and cellular miRNAs are protected within EVs.


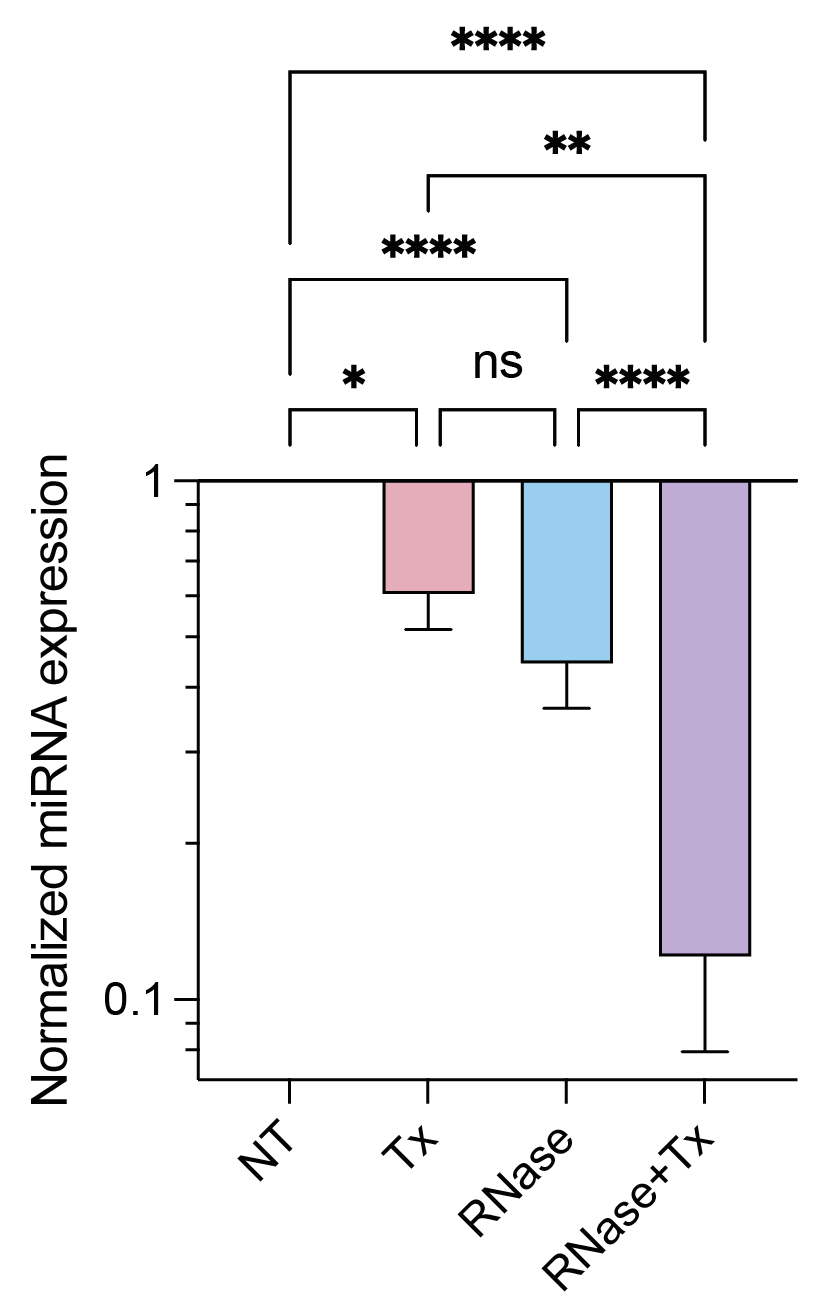


Four independent EV preparations (2 uninfected and 2 HCMV-infected) were subjected to RNase protection assay (NT: non-treated condition; Tx: Triton-X100; RNase: RNase treatment; RNase+Tx: both). For each experiment, two cellular miRNAs and one viral miRNA (if applicable) were quantified by RT-qPCR upon RNA extraction, and their level of expression were compared to the NT condition. Histogram represents the mean ± SEM of four independent experiments, in which the mean of expression was calculated from 2 or 3 quantified miRNAs and compared to the NT condition, which was set to 1. Statistical analysis was performed using a two-way ANOVA for which the “treatment” variable was significant (p<0.0001; “EV preparation” and “subject” were non-significant). A Tukey's multiple comparison test was carried out, with results indicated on the histogram (ns: non-significant; *: p<0.05; **: p<0.005; ****: p<0.0001).

## Supplementary Table S5. Proteins differentially expressed in NSCs upon differentiation depending on the infection status of placental sEVs.

The value of Log2 FoldChange (log2FC) is expressed for NSCs incubated with placental sEVs from HCMV-infected HIPECs compared to sEVs from uninfected HIPECs (HCMV *vs* NI).

## Supplementary Table S6. List of viral miRNAs found in placental sEVs upon HCMV infection.

## Supplementary Table S7. List of cellular miRNAs differentially expressed in placental sEVs upon HCMV infection.

## Supplementary Table S8. Integrated analysis of dysregulated miRNAs and their targets across proteomic and RT² datasets.

The table lists miRNAs significantly dysregulated in EVs from HCMV-infected placental cells (|log₂FC|≥2, p<0.05), together with their validated (miRTarBase) or predicted (TargetScan) target genes identified among significantly dysregulated (p<0.1) mRNAs (RT² array) and proteins (proteomic dataset) in recipient NSCs (script: https://github.com/CMart217/Scripts_EV_pla_differential_expression_of_miRNA.git). For each interaction, the corresponding dataset and database are indicated, as well as literature references (PMID) and prediction scores when available (NA: not available). Color code (EV CMV vs EV NI comparison): red = upregulated miRNAs; blue = downregulated miRNAs; green = upregulated targets; pink = downregulated targets; black = targets not significantly modulated. Inverse expression trends (miRNA up/down vs. target down/up) highlight potential regulatory relationships consistent with miRNA-mediated repression.
